# Nanofunctionalized Microparticles for Glucose Delivery in Three-Dimensional Cell Assemblies

**DOI:** 10.1021/acsami.4c02321

**Published:** 2024-04-02

**Authors:** Maria G. Fois, Aygul Zengin, Ke Song, Stefan Giselbrecht, Pamela Habibović, Roman K. Truckenmüller, Sabine van Rijt, Zeinab N. Tahmasebi Birgani

**Affiliations:** Department of Instructive Biomaterials Engineering, MERLN Institute for Technology-Inspired Regenerative Medicine, Maastricht University, P.O. Box 616, 6200 MD Maastricht, The Netherlands

**Keywords:** 3D cell culture, spheroids, drug delivery, microparticles, mesoporous silica nanoparticles, glucose

## Abstract

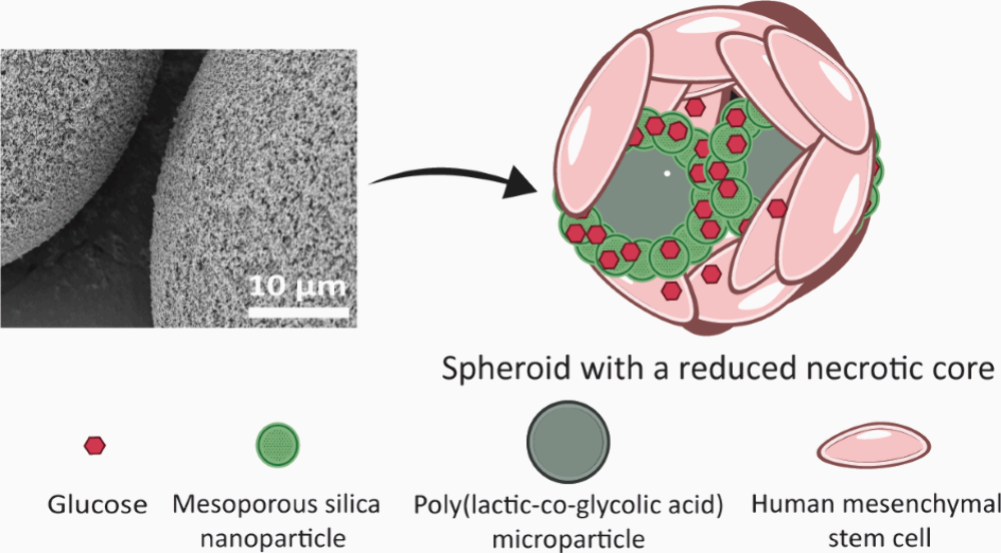

Three-dimensional (3D) cell assemblies, such as multicellular spheroids, can be powerful biological tools to closely mimic the complexity of cell–cell and cell–matrix interactions in a native-like microenvironment. However, potential applications of large spheroids are limited by the insufficient diffusion of oxygen and nutrients through the spheroids and, thus, result in the formation of a necrotic core. To overcome this drawback, we present a new strategy based on nanoparticle-coated microparticles. In this study, microparticles function as synthetic centers to regulate the diffusion of small molecules, such as oxygen and nutrients, within human mesenchymal stem cell (hMSC) spheroids. The nanoparticle coating on the microparticle surface acts as a nutrient reservoir to release glucose locally within the spheroids. We first coated the surface of the poly(lactic-*co*-glycolic acid) (PLGA) microparticles with mesoporous silica nanoparticles (MSNs) based on electrostatic interactions and then formed cell-nanofunctionalized microparticle spheroids. Next, we investigated the stability of the MSN coating on the microparticles’ surface during 14 days of incubation in cell culture medium at 37 °C. Then, we evaluated the influence of MSN-coated PLGA microparticles on spheroid aggregation and cell viability. Our results showed the formation of homogeneous spheroids with good cell viability. As a proof of concept, fluorescently labeled glucose (2-NBD glucose) was loaded into the MSNs at different concentrations, and the release behavior was monitored. For cell culture studies, glucose was loaded into the MSNs coated onto the PLGA microparticles to sustain local nutrient release within the hMSC spheroids. *In vitro* results demonstrated that the local delivery of glucose from MSNs enhanced the cell viability in spheroids during a short-term hypoxic culture. Taken together, the newly developed nanofunctionalized microparticle-based delivery system may offer a versatile platform for local delivery of small molecules within 3D cellular assemblies and, thus, improve cell viability in spheroids.

## 1 Introduction

Tissue engineering (TE) aims to develop functional replacements for lost or damaged tissues/organs. In particular, bottom-up TE aims at designing more sophisticated tissue constructs mimicking the complexity and hierarchical organization of native tissues.^1−3^ This approach mainly relies on the concept of self- or directed assembly of individual microunits with defined spatial and temporal properties to create larger structures.^4^ In this context, cellular spheroids have captured great interest as building blocks of tissue-like constructs since they exhibit physiological cell–cell/-matrix interactions and can mimic gradients of soluble factors present in native tissues.^5,6^ However, a major limitation of these three-dimensional (3D) cell assemblies is the lack of internal vascular networks, which leads to insufficient oxygen and nutrient transport. This influences the viability of larger spheroids (diameter >200 μm), which show a necrotic core in a size-dependent manner, due to the diffusion limit of oxygen and nutrients in tissues (100–200 μm).^5,7^

Recently, the internal incorporation of cell-sized microparticles into spheroids has been used to guide cellular processes such as proliferation and differentiation.^2,6^ The microparticles can provide many functions, such as acting as synthetic spacers in between the cells in spheroids to regulate oxygen and nutrient transport.^8,9^ For example, Hayashi et al. combined gelatin hydrogel microspheres with rat bone marrow mesenchymal stem cells (MSCs) to form cell-biomaterial aggregates.^10^ The authors reported that the presence of the gelatin microspheres within the aggregates enhanced the oxygen permeability, thus leading to the improvement of cell adhesion, proliferation, and osteogenic differentiation. Furthermore, it has been shown that microparticles can deliver several types of biochemical molecules, such as growth factors^11^ or adhesion proteins,^12^ to regulate cell behavior within cell spheroids. In these cases, the chemical composition and microstructure of the microparticles, as well as their interaction with the loaded molecules, were determinants of the controlled delivery of the molecules. Biochemical surface functionalization of microparticles with, for example, drugs, growth factors, proteins and peptides, nutrients and antibodies, has been widely used to orchestrate the cellular response to microparticles, for example, in terms of cell attachment, proliferation and migration.^13^

It is known that nanoparticles are excellent candidates as effective delivery systems for different cargos. Nanoparticles can enhance the stability and solubility of their cargo^14^ and allow for high loading efficacy.^15^ In addition, the nanoparticles high surface-to-volume ratio and unique physical and chemical properties^16−18^ can improve or guide cell adherence. Hence, functionalizing the microparticle surface with nanoparticles can improve their effectivity as drug delivery systems, as the nanoparticles act as the cargo reservoirs and enhance the cellular response to the microparticles. For example, Na et al. coated poly(lactic-*co*-glycolic acid) (PLGA) microparticles with heparin-poly(l-lysine) nanoparticles based on layer-by-layer assembly for stem cell therapy. They reported that composite systems of the MSCs and the functionalized microcarriers enhanced cell adhesion, growth, and differentiation compared to only PLGA microparticles.^19^ In addition, they were also able to release transforming growth factor (TGF)-β 3 from heparin-poly(l-lysine) nanoparticle coatings on PLGA microparticles to induce neocartilage formation.^20^

Among the various types of nanoparticles, mesoporous silica nanoparticles (MSNs) are promising drug delivery vehicles due to their high biocompatibility, stable mesoporous structure, tunable pore size, and easy surface functionalization.^21^ Previous studies have shown that MSNs can effectively carry a wide range of cargoes such as anticancer drugs,^22^ genes (siRNA),^23^ bioinorganic ions,^24^ and small molecules, such as dexamethasone.^25^ In tissue engineering applications, MSNs have been utilized to function as delivery vehicles and as part of matrix-mimicking scaffolding biomaterials, at the same time.^26^ However, the use of MSNs in combination with cell spheroids has mostly been limited to cancer nanomedicine,^27−29^ as they can release anticancer drugs intracellularly*.*

**Figure 1:**
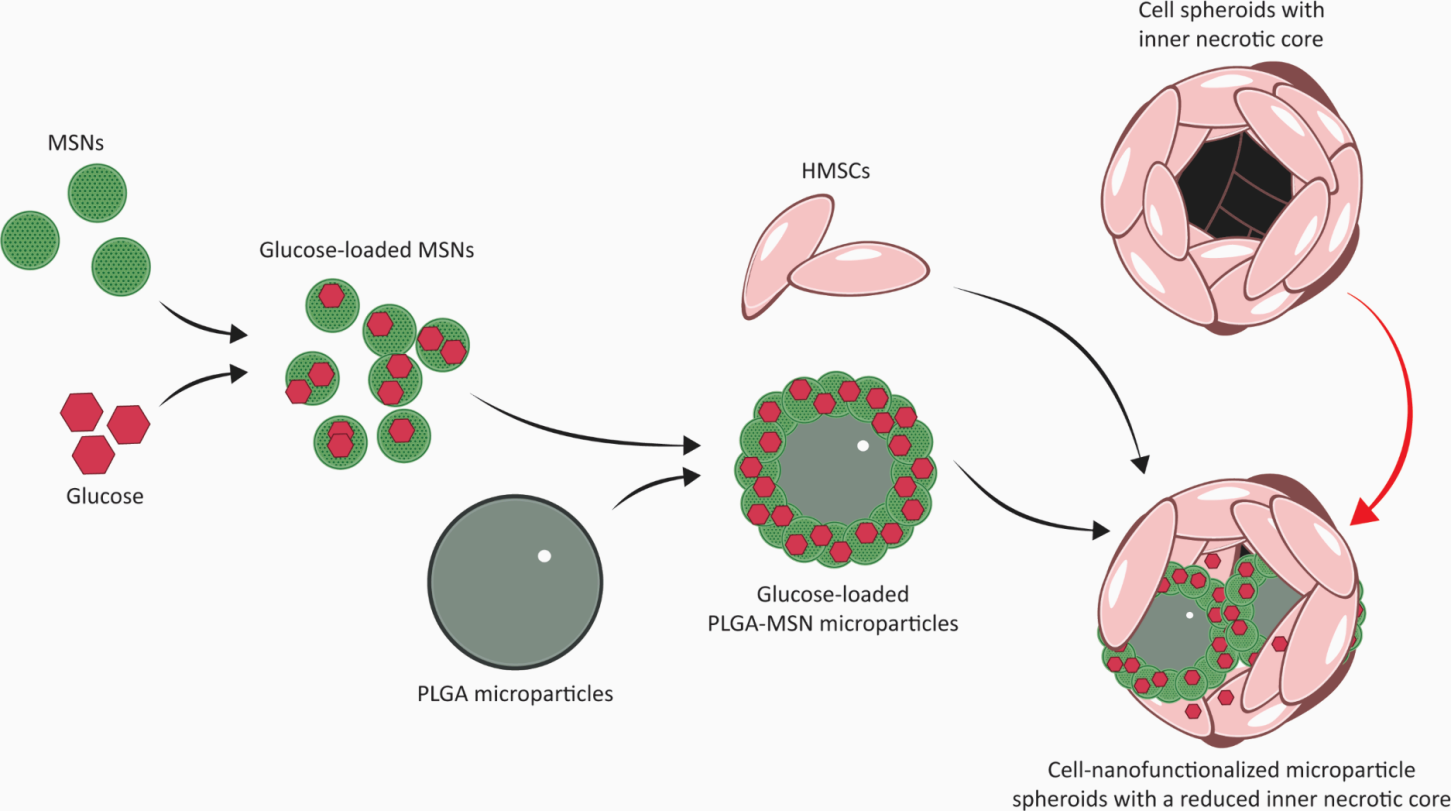
Schematic illustration of the hierarchal structure and components of the cell-nanofunctionalized microparticle spheroids to reduce spheroids’ inner necrotic cores. From the left: Glucose is loaded into the pores of MSNs, and PLGA microparticles are coated with glucose-loaded MSNs. The glucose-loaded PLGA-MSN microparticles are coaggregated with human mesenchymal stem cells (hMSCs) to form cell-nanofunctionalized microparticle spheroids. The PLGA-MSN microparticles are cell-organized within the spheroid and act as multipoint delivery centers to the cells. In this way, the cells within the spheroids are directly exposed to glucose released from the MSNs, which prevents the formation of an inner necrotic core.

In this study, we present a new strategy based on hybrid nanofunctionalized microparticle biomaterials using PLGA microparticles as a core material and MSNs as functional nanocoatings on microparticles for local delivery of nutrients to modulate and improve the biological performance of cellular spheroids, particularly in terms of viability and metabolic activity. Here, PLGA microparticles act as nutrient local delivery centers between the cells and MSNs as reservoirs for nutrients. Our proposed design relies on coating the negatively charged PLGA microparticle surface with positively charged MSNs based on electrostatic interactions and then creating cell-nanofunctionalized microparticle spheroids (Figure 1). We selected PLGA microparticles since PLGA is Food and Drug Administration (FDA)-approved, biocompatible, and biodegradable.^30,31^ Moreover, PLGA microparticles have been widely applied as cell carriers and drug delivery vehicles in TE and regenerative medicine applications.^19,30,32^ In our MSN-coated PLGA (PLGA-MSN) microparticle-based delivery system, the PLGA microparticles function as synthetic spacers and cell-size 3D substrates for the MSN coating. On the other hand, the MSNs provide high surface area that can be used for loading high content of nutrients. As a proof of concept, we chose glucose as a reference small molecule for local release from the MSN coating. Glucose has been previously described as an essential moiety for MSCs survival upon transplantation, which prolonged MSCs viability in low oxygen environments.^33^

To develop the cell-nanofunctionalized microparticle spheroids, we first evaluated the stability of the MSN coating on the microparticle surface under cell culture conditions. Then, we evaluated the spheroid formation and viability in the presence of the designed PLGA-MSN microparticles. Afterward, we loaded fluorescently labeled glucose into the MSNs with different loading concentrations to examine the release behavior with a fluorescence spectrometer. The viability and metabolic activity of the cells in cell-nanofunctionalized microparticle spheroids with and without loaded glucose were assessed in normal oxygen conditions and, to further investigate the performance of the proposed delivery system in a harsher culture environment, also in hypoxic conditions. In addition, we studied the potential of nanofunctionalized microparticles in reducing necrotic regions in large spheroids. The proposed delivery strategy based on the newly developed nanofunctionalized microparticles may represent a smart solution to locally deliver nutrients to the inner cores of spheroids to reduce cell death and improve cell survival in spheroids.

## 2 Methods

### 2.1 MSN Synthesis and Characterization

MSNs were functionalized with free amine groups on their surface and free thiol groups in their core as we similarly reported previously.^34^ In short, a mixture of 14.3 g of triethanolamine (TEA; Sigma-Aldrich), 1.63 g of tetraethyl orthosilicate (TEOS; Sigma-Aldrich), and 112 mg of 3-mercaptopropyl triethylsilane (MPTES; Sigma-Aldrich) was heated at 90 °C for 20 min under static conditions (“Mixture 1”). A solution of 2.7 mM ammonium fluoride (AF; Sigma-Aldrich) and 2.41 mL of cetyltrimethylammonium chloride (CTAC; Sigma-Aldrich) in 21.7 g of Milli-Q water was heated at 60 °C for 20 min (“Mixture 2”). Mixture 2 was rapidly added to Mixture 1, and the resulting mixture was stirred for 20 min at room temperature (RT). Afterward, 138.2 mg of TEOS was added in four equal increments every 3 min. After 30 min of stirring at RT, 19.3 mg of TEOS and 20.5 mg of 3-aminopropyl triethoxysilane (APTES; Sigma-Aldrich) were added to the mixture and left stirring overnight at RT. The particles were collected by 20 min centrifugation at 7800 rpm and washed once with absolute ethanol (Sigma-Aldrich). For organic template extraction, the particle suspension was heated at 90 °C for 45 min under reflux in a 20 g/L ammonium nitrate (Sigma-Aldrich) solution in ethanol. MSNs were collected by centrifugation and washed with absolute ethanol before the second template extraction in 100 mL of a 3.7% v/v hydrochloric acid solution (HCl; Sigma-Aldrich) in ethanol for 45 min at 90 °C. After two washing steps with absolute ethanol, the MSN suspension was stored at −20 °C in absolute ethanol.

The hydrodynamic diameter and zeta potential of synthesized MSNs suspended in Milli-Q water (0.5 mg/mL) were determined by dynamic light scattering (DLS; Zetasizer Nano Sysmex, Malvern Panalytical). The shape and porosity of the nanoparticles were imaged by using scanning electron microscopy (SEM; Teneo, FEI) and transmission electron microscopy (TEM; FEI Tecnai). For SEM imaging, the samples were prepared by placing a drop of nanoparticle solution on aluminum stubs and then drying at RT. Later, the samples were sputter-coated (Q150T ES, Quorum Technologies) with a thin layer of iridium. SEM imaging was operated at an accelerating voltage of 2 kV and working distance of 2 mm. For TEM imaging, MSN suspension in ethanol was dropped on a TEM carbon grid and imaged after air-drying at RT. The size of nanoparticles based on TEM images was determined via image analysis by using ImageJ 1.53t^35^ and shown as mean ± standard deviation.

### 2.2 Preparation and Characterization of MSN-Coated PLGA Microparticles

PLGA microparticles (average diameter = 50 μm; lactide-*co*-glycolide ratio of 50/50; Sigma-Aldrich) were first treated by oxygen plasma for 5 min at 140 W (Diener electronic Femto) and their surface charge was measured via a ZetaSizer Nano (Malvern Instruments, Sysmex). In addition, the PLGA microparticles before and after plasma treatment were inspected by SEM (JSM-IT200, Jeol) at an accelerating voltage of 10 keV. The microparticles were coated with a thin layer of gold by using a sputter-coater (SC7620, Quorum) prior to SEM imaging. Plasma-treated PLGA microparticles and MSNs were dispersed separately in 500 μL of Milli-Q water (pH 7.4). Then, the PLGA solution was slowly added to the MSN solution. The mixture was sonicated and vortexed for a few seconds and left overnight under shaking at 600 rpm at RT. The mixture was centrifuged at 400 rpm to separate unattached MSNs from PLGA-MSN microparticles. After centrifugation, the PLGA-MSNs formed a pellet at the bottom of an Eppendorf tube, while unattached MSNs remained in the supernatant. The supernatant was discarded, and fresh Milli-Q water was added to resuspend the pellet. This step was repeated at least six times to remove unattached MSNs completely. Lastly, PLGA-MSNs were dispersed in fresh Milli-Q water. To show the versatility of our coating method, we also coated poly(methyl methacrylate) (PMMA; Cospheric) and polyethylene (PE; Cospheric) microparticles with MSNs as described above to obtain PMMA-MSN and PE-MSN microparticles, respectively.

The MSN coating on PLGA microparticles was confirmed by using SEM imaging and fluorescence microscopy (ECLIPSE Ti, Nikon Instruments). For SEM imaging, the samples were sputter-coated with a thin iridium layer as described earlier. For fluorescence imaging, the MSNs were core-labeled with ATTO647N-Maleimide dye (Atto-TEC). A 10 mg/mL solution of MSNs in absolute ethanol was fluorescently labeled by adding a 1:200 dilution of the ATTO647N dye solution. The mixture was stirred overnight in the dark. Then, the MSNs were collected by centrifugation (14000 rpm, 20 min) and washed once with absolute ethanol. The MSNs were resuspended in absolute ethanol at a concentration of 10 mg/mL. The fluorescently labeled MSNs were used for coating PLGA microparticles. The coated microparticles were visualized by using a fluorescence microscope (ECLIPSE Ti, Nikon Instruments) after 1 day and 9 days of incubation in Milli-Q water at RT.

### 2.3 PLGA-MSN Stability in Cell Culture Media

The PLGA-MSNs were incubated in Dulbecco′s modified Eagle′s medium (DMEM; Sigma-Aldrich) supplemented with 100 U/mL penicillin and 100 μg/mL streptomycin (Thermo Fisher Scientific, Gibco), without and with addition of 10% v/v fetal bovine serum (FBS, Sigma-Aldrich), over 14 days at 37 °C. At days 2, 7, and 14, samples (*n* = 1) were centrifuged at 500 rpm. Then, the cell culture media was discarded, and the PLGA-MSN pellet was dispersed in Milli-Q water (1 mg/mL). For SEM imaging, aluminum SEM stubs were covered with double-sided carbon tape. A drop of PLGA-MSN suspension was added to the tape and left to dry overnight*.* Before imaging, the samples were sputter-coated with a thin layer of iridium or gold. SEM imaging was operated at an accelerating voltage of 2 kV and working distance of 6 mm for experiments in cell medium without FBS supplementation (Teneo, FEI) and at an accelerating voltage of 10 kV for experiments in cell medium with FBS supplementation (JSM-IT200, Jeol). For the latter, the elemental composition of the microparticles was also assessed at each time point using energy-dispersive X-ray spectroscopy (EDS). The stability assays for PE-MSNs and PMMA-MSNs microparticles were performed in the same manner after 7 days in a cell culture medium without FBS supplementation at 37 °C.

### 2.4 Fabrication, Characterization, and Preparation of the Microwell Arrays

Arrays of circular U-bottom microwells from polycarbonate (PC) were fabricated via gas-assisted microthermoforming as previously described.^36,37^ Briefly, an array of 30 blind holes (5 rows by 6 columns), hexagonally arranged, was micromilled on a brass mold. This brass mold served as a template for the microscale thermoforming of PC films to form microwell arrays. In contact with the brass mold, a 50 μm-thick PC film was clamped into a heated press, between the brass mold and a brass counter plate. The mold’s geometrical features were replicated into the PC film by softening the film at a temperature of 154 °C and 3D stretching it by a gas pressure of 20 bar.

Visual inspection of the microwell arrays was performed via SEM imaging (JSM-IT200, JEOL) at various magnifications using an accelerating voltage of 10 kV and working distance of 10 mm. To this end, the arrays were sputter-coated with a thin layer of gold (SC7620, Quorum Technologies). Quantitative characterization of microwells’ geometrical features, including their vertical depth, was done using confocal laser scanning profilometry (VK-X200, Keyence).

Thermoformed microwell arrays were mounted at the bottom of the wells of 96-well plates with the aid of O-rings (ERIKS). An isopropanol dilution series (100%, 70%, 50%, 20%, and 0% v/v in Milli-Q water) was performed to wet, sterilize, and wash the microwells as well as the O-rings. One day before cell seeding, 100 μL of 1% w/v Pluronic F108 (Sigma-Aldrich) was dispensed onto each microwell array and incubated overnight at 37 °C to form a nonadherent coating on the surface of the PC microwells.

### 2.5 HMSC Culture and Formation of hMSC-Nanofunctionalized Microparticle Spheroids

HMSCs at passage 0 were purchased from Lonza (cat: PT-2501, lot: 19TL329433), expanded according to manufacturer instruction, subcultured until passage 2, and kept frozen as previously described by our former lab.^38^ Cells at passage 3 were seeded in tissue culture plastic flasks at a density of 2000 cells/cm^2^ in a basic medium and incubated at 37 °C under 5% CO_2_. The basic medium was DMEM-supplemented with 1 mM sodium pyruvate (Gibco), 10% FBS (Sigma-Aldrich, lot BCBX5318), 0.2 mM 2-phosphate sesquimagnesium salt hydrate (Sigma-Aldrich), and 100 U/mL penicillin and 100 μg/mL streptomycin (Thermo Fisher Scientific, Gibco). Once about 75% confluence was reached, the cells were trypsinized and resuspended in a fresh basic medium.

On the day of the experiment, about 3 μg of biomaterial microparticles, PLGA and PLGA-MSN, were resuspended in Dulbecco’s phosphate buffered solution (DPBS; Sigma-Aldrich) and dispensed into microwell arrays. Once the biomaterial microparticles settled into the microwells, the DPBS was removed. HMSCs were seeded into the microwells at a density of approximately 5000 cells per microwell and incubated at 37 °C under 5% CO_2_ for at least 2 h. The medium was then gently topped up and refreshed twice a week. Spheroids of solely hMSCs served as controls in all experiments. For obtaining larger spheroids, about 6 μg of biomaterial microparticles, PLGA and PLGA-MSN, were dispensed into the microwell arrays and coseeded with approximately 10000 cells per microwell as described above.

### 2.6 Assessment of Morphology of hMSC-Nanofunctionalized Microparticle Spheroids

Qualitative assessment of the morphology of the hMSC-nanofunctionalized microparticle spheroids was performed via SEM (JSM-IT200, JEOL). After 3 days in culture, the spheroids were fixed with warm 10% formaldehyde (FA; Sigma-Aldrich) in DPBS for 20 min. After this, the spheroids were dehydrated in an ethanol solution series (30%, 50%, 70%, 80%, 90%, 96%, and 100% v/v ethanol in Milli-Q water). The samples were incubated in each dilution for 15 min, and after that, an additional incubation in hexamethyldisilazane for 15 min was performed. Once dried, the samples were imaged by SEM using an accelerating voltage of 10 kV and working distance of 10 mm. Prior to imaging, the spheroid samples were sputter-coated with a thin layer of gold (Quorum Technologies, SC7620).

### 2.7 Immunocytochemical Analysis of Focal Adhesions in hMSC-Nanofunctionalized Microparticle Spheroids

Formation and distribution of focal adhesions in hMSC-nanofunctionalized microparticle spheroids were qualitatively assessed via confocal fluorescence microscopy analysis. After 4 and 24 h in culture, the spheroids were fixed with warm 10% FA in DPBS solution for 20 min and permeabilized using a 0.1% v/v solution of Triton-X100 (Sigma-Aldrich) in Milli-Q water for 30 min. After this, an unspecific binding blocking step of 1 h was performed in a casein-based blocking serum (CAS-Block Histochemical Reagent; Thermo Fisher Scientific). The spheroids were then incubated overnight with an Alexa Fluor 647-conjugated monoclonal antivinculin antibody (ab196579, Abcam) solution in a 0.1% w/v bovine serum albumin (BSA; VWR) solution in phosphate-buffered saline (PBS) with a 1:100 dilution to visualize focal adhesions. At the same time, cell nuclei were counterstained overnight using diamidino-2-phenylindole dihydrochloride (DAPI; Sigma-Aldrich) at a dilution of 1:200 in 0.1% BSA in PBS. The imaging was performed using a confocal laser scanning electron microscope (TCS SP8 STED, Leica Microsystems), acquiring z-stacks of 4 μm thickness for each image.

### 2.8 Quantification of the Size of hMSC-Nanofunctionalized Microparticle Spheroids

The size of the hMSC-nanofunctionalized microparticle spheroids was quantified via image analysis of confocal fluorescence microscopy images after 3 days of culture. At day 3 of the culture, the spheroids were fixed and permeabilized as described above. Subsequently, an unspecific binding blocking step was performed as described above. Cytoskeletal F-actin and cell nuclei in the spheroids were stained with Alexa Fluor 568 Phalloidin (Thermo Fisher Scientific, Invitrogen) and DAPI, respectively, both at a dilution of 1:200 in 0.1% BSA in PBS overnight and protected from the light. Prior to imaging, the samples were washed twice with PBS for 30 min. The imaging was performed using a confocal laser scanning electron microscope (TCS SP8 STED, Leica Microsystems), acquiring z-stacks of 4 μm thickness for each image.

To measure the size of the spheroids in terms of the projected area, the maximum projection images of the phalloidin staining were composed in ImageJ and the total area of the phalloidin channel was quantified in terms of total pixel area using CellProfiler 4.2.1 (https://cellprofiler.org/).

### 2.9 2-NBD Glucose Loading

To monitor the glucose release from the pores of MSNs (not coated on microparticles), we used fluorescently labeled 2-NBD glucose (Glu_F_; Promega). The MSNs were dispersed in solutions of Glu_F_ in Milli-Q water with 1, 10, or 100 mM Glu_F_ concentrations and kept under steering at 900 rpm, overnight at RT. The mixture was then centrifuged, and the supernatant, containing excess Glu_F_, was discarded. The pellets of Glu_F_-loaded MSNs were dispersed in Milli-Q water (10 mg/mL) and 500 μL of this suspension was added to a mini dialysis tube (Slide A analyzer, Thermo Fischer). The tubes were attached inside a UV cuvette, which was filled with Milli-Q water (3.5 mL). The fluorescence measurements were performed using a spectrometer (Cary Eclipse Fluorescence Spectrometer, Agilent) at excitation and emission wavelengths of 465 and 540 nm, respectively. The measurements were performed every 15 min for the first 3 h and every hour for 3 days at RT.

For Glu_F_ loading into pores of PLGA-MSNs, the PLGA-MSNs were left overnight shaking in either 1 mM (PLGA-MSN (1 mM Glu_F_)) or 100 mM Glu_F_ solution (PLGA-MSN (100 mM Glu_F_)) at 900 rpm. The mixture was then centrifuged, and the excess Glu_F_ solution was discarded. Later, Glu_F_-loaded PLGA-MSNs were dispersed in fresh Milli-Q water. The release of Glu_F_ from PLGA-MSNs was monitored as described for the Glu_F_-loaded MSNs. For cell culture experiments, PLGA-MSNs were left shaking overnight in 1 and 100 mM d-glucose, simply referred to as “glucose” throughout the text, solution (Glu; Sigma-Aldrich) and denoted as “PLGA-MSN (1 mM Glu)” and “PLGA-MSN (100 mM Glu)”, respectively.

### 2.10 Cell Metabolic Activity in hMSC-Nanofunctionalized Microparticle Spheroids

The metabolic activity, i.e., the production of adenosine triphosphate (ATP), of hMSCs in spheroids was quantified using a CellTiter-Glo 3D Assay (Promega) kit according to manufacturer instructions. Quantification of metabolic activity within the spheroids was performed on two occasions during this study. Initially, metabolic activity was quantified in DMEM after 3 days of culture for solely hMSC, hMSC-PLGA, and hMSC-PLGA-MSN spheroids. Further in the investigation, metabolic activity of hMSCs within the spheroids was quantified after 1, 3, and 7 days of culture in DPBS in both conditions of normoxia, and hypoxia (5% pO_2_), comparing solely hMSC, hMSC-PLGA-MSN, and hMSC-PLGA-MSN (1 mM Glu) and hMSC-PLGA-MSN (100 mM Glu) spheroids.

### 2.11 Visualization and Quantification of Dead cells in hMSC-Nanofunctionalized Microparticle Spheroids

The quantification of dead cells within the hMSC-nanofunctionalized microparticle spheroids was performed after 1 and 7 days of culture via image analysis of confocal fluorescence microscopy images in order to determine the cell viability in spheroids. To this end, hMSC-nanofunctionalized microparticle spheroids were cultured in no glucose DMEM (DMEM no Glu; Gibco) in both conditions of normoxia and hypoxia (5% pO_2_). HMSC spheroids cultured in three different media, DMEM no Glu, DMEM, and high glucose DMEM (DMEM high Glu; Gibco), served as references. At days 1 and 7 of culture, before the spheroid fixation, the dead cells within the spheroids were marked with a LIVE/DEAD Fixable Far-Red Dead Cell Stain Kit (Thermo Fisher Scientific, Invitrogen). After this, the samples were fixed, permeabilized, and blocked as previously described. F-actin cytoskeleton and cell nuclei were counterstained as described above.

For the larger hMSC-nanofunctionalized microparticle spheroids, the quantification of dead cells was performed after 3 days of culture in normoxia in the same manner. In this case, hMSC-PLGA-MSN and hMSC-PLGA-MSN (100 mM Glu) spheroids were cultured in no glucose DMEM. HMSC spheroids with no microparticles were cultured in normal and no glucose DMEM.

The spheroids were imaged using a confocal laser scanning electron microscope (TCS SP8 STED, Leica Microsystems), acquiring z-stacks of 4 μm thickness for each image. For each of the above conditions, images of spheroids (*n* = 4 and 6 spheroids for small and large spheroids, respectively) were quantified for dead cells. Maximum projection images of the hMSC-nanofunctionalized microparticle spheroids were composed in ImageJ and the quantification of dead cells was performed on these images using CellProfiler, by identifying the colocalization loci of the cell nuclei and dead cells.

### 2.12 Statistical Analysis

All biological experiments were performed with *n* = 3 replicates/triplicates unless indicated otherwise in the text. Results were expressed as the mean ± standard deviation and graphically represented as error bars. Statistical analysis was performed in Graphpad Prism 8.3 using a one- or two-way analysis of variance (ANOVA) followed by Tukey’s post hoc test for multiple comparisons. “*”, “**”, “***”, and “****” indicate statistically significant differences with *p* values smaller than 0.05, 0.01, 0.001, and 0.0001, respectively. “n.s.” stands for “not statistically significant”.

## 3 Results and Discussion

### 3.1 Coating of PLGA, PMMA, and PE Microparticle Surfaces with MSNs via Electrostatic Interactions

**Figure 2:**
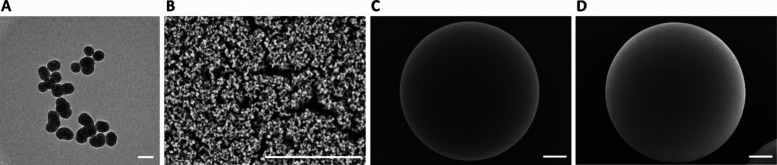
Nano- and microparticle visualization. (A) TEM image of MSNs. Scale bar represents 100 nm. (B) SEM image of MSNs. Scale bar represents 4 μm. (C) SEM image of bare PLGA microparticles before oxygen plasma treatment. Scale bar represents 10 μm. (D) SEM image of oxygen plasma-treated, bare PLGA microparticles. Scale bar represents 10 μm.

In this study, we coated PLGA microparticles with glucose-loaded MSNs to allow the local release of glucose into hMSC spheroids. Here, we chose to develop a hybrid system with PLGA microparticle cores coated with MSNs. PLGA microparticles were chosen to support spheroid formation and MSNs to allow efficient nutrient delivery, owing to their ordered porous structure and high surface area. Using MSNs in the form of a coating also aimed to avoid their internalization by cells^39^ during coaggregation and spheroid formation. First, core–shell functionalized MSNs were synthesized as described earlier with minor modifications.^34^ Specifically, these MSNs have free thiol groups in their core to allow further fluorescent labeling and free amine groups on their surface to promote electrostatic interactions with the PLGA surface. The obtained MSNs showed a uniform spherical morphology and porous structure with an average diameter of 78.2 ± 6.85 nm, as observed by TEM and SEM (Figure 2A,B). The DLS results demonstrated that the average hydrodynamic diameter of MSNs was 240.9 ± 3.26 nm, which was larger than the diameter observed by TEM under dry conditions. This can be attributed to the hydration layer formed around the nanoparticle surface when suspended under aqueous conditions. The polydispersity index of MSNs was 0.268, which indicates a narrow size distribution and a homogeneous sample. This was further evidenced by TEM imaging (Figure 2A).

**Figure 3:**
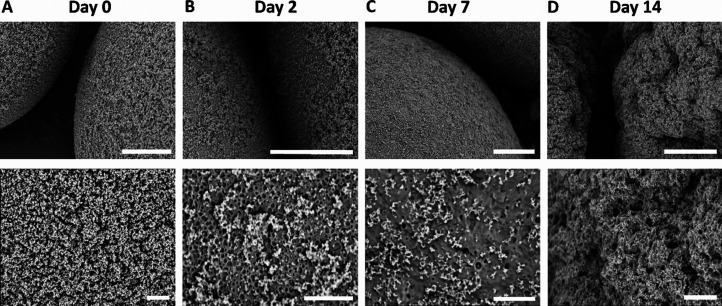
Stability of PLGA-MSN microparticles in cell culture medium over the course of 14 days at 37 °C. SEM images of PLGA-MSN microparticles (A) before incubation (day 0) and after (B) 2, (C) 7, and (D) 14 days of incubation. Scale bars represent (top row) 10 μm and (bottom row) 2 μm.

Zeta potential measurements showed that the MSNs were positively charged (40.2 ± 0.40 mV) when measured in Milli-Q water, while the PLGA microparticles were negatively charged with a Zeta potential of −10.1 ± 1.97 mV. To create a strong interaction between the micro- and nanoparticles and allow effective, stable MSN coating onto the PLGA surface, we first treated the PLGA microparticles with oxygen plasma to introduce polar groups, such as hydroxyl and carboxyl, to the surface of the microparticles^40,41^ and further decrease their negative surface charge. The zeta potential of the bare PLGA microparticles changed to −17.1 ± 3.35 mV after plasma treatment. Plasma treatment did not alter the PLGA surface morphology (Figures 2C and D). Negatively charged plasma-treated PLGA microparticles were then coated with positively charged MSNs based on electrostatic interactions. SEM images showed a homogeneous and complete coverage of the PLGA microparticles with MSNs (Figure 3A). Fluorescence microscopy images further confirmed the uniform distribution of fluorescently labeled MSNs on the microparticle surfaces (Figure S1). Although it has been previously shown that PLGA microparticles could be successfully coated with polyethylenimine (PEI) to create a positively charged PLGA surface,^42^ this is the first time that MSNs were used as a nanocoating on a microparticle surface. Compared to similar studies reporting on nanoparticle coating of PLGA microparticles,^19,20^ substantially denser nanoparticle coatings on PLGA microparticles were obtained in our approach using MSNs. This might be explained by inherent properties of MSNs, and different pretreatment processes of the PLGA microparticles. In our approach, we enhanced the amount of charged groups on the PLGA surface by oxygen plasma treatment instead of directly coating microparticles via positively charged polymers such as PEI.

To investigate whether a microparticle coating with MSNs could be extended to different types of biomaterials, we also coated the surfaces of PMMA and PE microparticles. After plasma treatment, the zeta potentials of PMMA and PE microparticles were −42.7 ± 1.24 and −7.28 ± 1.49 mV, respectively. We also visualized the surface features of bare PE and PMMA. The results showed that PE microparticles have smooth or slightly dimpled surfaces while PMMA has some small granules on their surface (Figure S2). The SEM images of PE-MSNs and PMMA-MSNs revealed that MSNs successfully coated the surfaces of these microparticles. However, the MSN coating density on PE microparticles was lower than that on PLGA microparticles (Figure S3). The reduced MSN coating density on PE microparticle surfaces may be attributed to the hydrophobic nature of the particles.^43^ In the case of PMMA microparticles, SEM images demonstrated a slightly patchy MSN coating (Figure S3). This might be due to the influence of small artifacts on the PMMA surface (Figure S2B). All in all, the MSN coating density on the microparticles is obviously significantly influenced by the surface properties of the substrate material.

### 3.2 Stability of the MSN Coating on the PLGA Microparticles

We initially examined the presence and stability of the MSN coating on PLGA microparticles with fluorescently labeled MSNs in Milli-Q water via fluorescence microscopy. The images revealed that MSN coating on the PLGA surface was still present after 9 days of incubation at RT (Figures S1A-C). In the next step, to obtain a deeper understanding of the behavior of the microparticles, specifically in physiological condition, the stability of the MSN coating on the PLGA microparticle surface was tested in cell culture medium without and with FBS supplementation at 37 °C over 14 days. SEM images showed that the 3D surface of PLGA microparticles was fully covered with MSNs on day 0 (Figure 3A). However, we observed the formation of pores on the PLGA microparticle surface on days 2 and 7 when incubated in cell culture medium without FBS supplementation (Figure 3B,C) and on day 7 when incubated in cell medium with FBS supplementation (Figure S4A,B). At day 14 in cell culture medium without and with FBS supplementation, the microparticles presented larger defects on the surface, indicating the degradation of PLGA (Figure 3D, Figure S4A,B). PLGA is known to be biodegradable; previous studies reported that PLGA has a varying degradation time, which is mainly influenced by its monomer composition (lactic acid/glycolic acid ratio) and molecular weight, ranging from approximately 4 weeks to 20 weeks in PBS at 37 °C.^44,45^ Especially, increased glycolic acid percentage in the polymer, e.g., in PLGA 50:50 (PLA/PGA), resulted in faster degradation than PLGA 75:25 due to improved hydrophilicity.^44^ Here, the microparticle composition (PLA/PGA 50:50), oxygen plasma pretreatment, and complexity of the cell culture medium may have led to faster microparticle degradation in cell culture medium both without and with FBS supplementation.

Furthermore, we observed that in cell culture medium without FBS addition, the nanocoating was partly present on the PLGA surface at day 7. The partial presence of the MSN coating might be due to the instability of charge-based interactions, and the degradation of the PLGA could be an additional reason that accelerated this process. In cell culture medium containing FBS, the presence of the MSNs on the PLGA surface was confirmed at all time points by the presence of silicon (Si) peaks in the EDS spectra of the PLGA-MSNs (Figure S4C). Here, protein adsorption on the PLGA-MSN surface may have improved the stability of the nanocoatings.

We also investigated the stability of MSN coatings on nondegradable microparticles from PE and PMMA in cell culture medium without FBS at 37 °C for 7 days. The results showed no significant loss of MSN coating on PE microparticles as a function of time, indicating good stability of the MSN coating on these 3D surfaces (Figure S3A,B). However, for PMMA microparticles, the coating became patchier over time (Figure S3C,D).

### 3.3 Microwell Arrays Support the Culture of hMSC-Nanofunctionalized Microparticle Spheroids

To allow the aggregation and maintenance of the spheroids over time, their culture was performed in 3D microwells. The use of thermoformed microwell arrays has been extensively reported to maintain spheroids,^36^ organoids,^46^ and other 3D cellular constructs^47,48^ in culture. Advantages of thermoformed microwell chips are the possibility to upscale fabrication and analysis in a high-throughput manner and apply patterned physical, chemical and biological surface or bulk modification.^37^ More important for this study, thermoformed microwells allow the culture of 3D microtissues (e.g., spheroids) in a standardized fashion in terms of their shape and size,^49^ which makes them suitable to obtain reproducible readouts when drugs and other soluble compounds are screened in 3D. The microwell arrays (Figure S5A,B) were obtained via gas-assisted microthermoforming of PC films.^36,37^ The microwells had an outer diameter of 549.75 ± 5.97 μm and vertical depth of 317.48 ± 66.10 μm at their apex. Here, PC was chosen because of its well-known characteristics of biocompatibility and transparency.^36^ The latter was optimal to investigate the spheroids via high-resolution imaging.

The PC surface of the microwell chips was coated with a thin layer of Pluronic F108 (Sigma-Aldrich), a poly(ethylene glycol)-*co*-poly(propylene glycol)-*co*-poly(ethylene glycol) (PEG–PPG-PEG) triblock copolymer to shield the absorption of proteins and therefore prevent cell attachment on the PC surface.^50^ This facilitated the formation and compaction of the cell-nanofunctionalized microparticle spheroids at the center of the round-bottom microwells.

### 3.4 Coseeding of hMSCs and Microparticles in PC Microwells Resulted in the Formation of Spheroids

**Figure 4:**
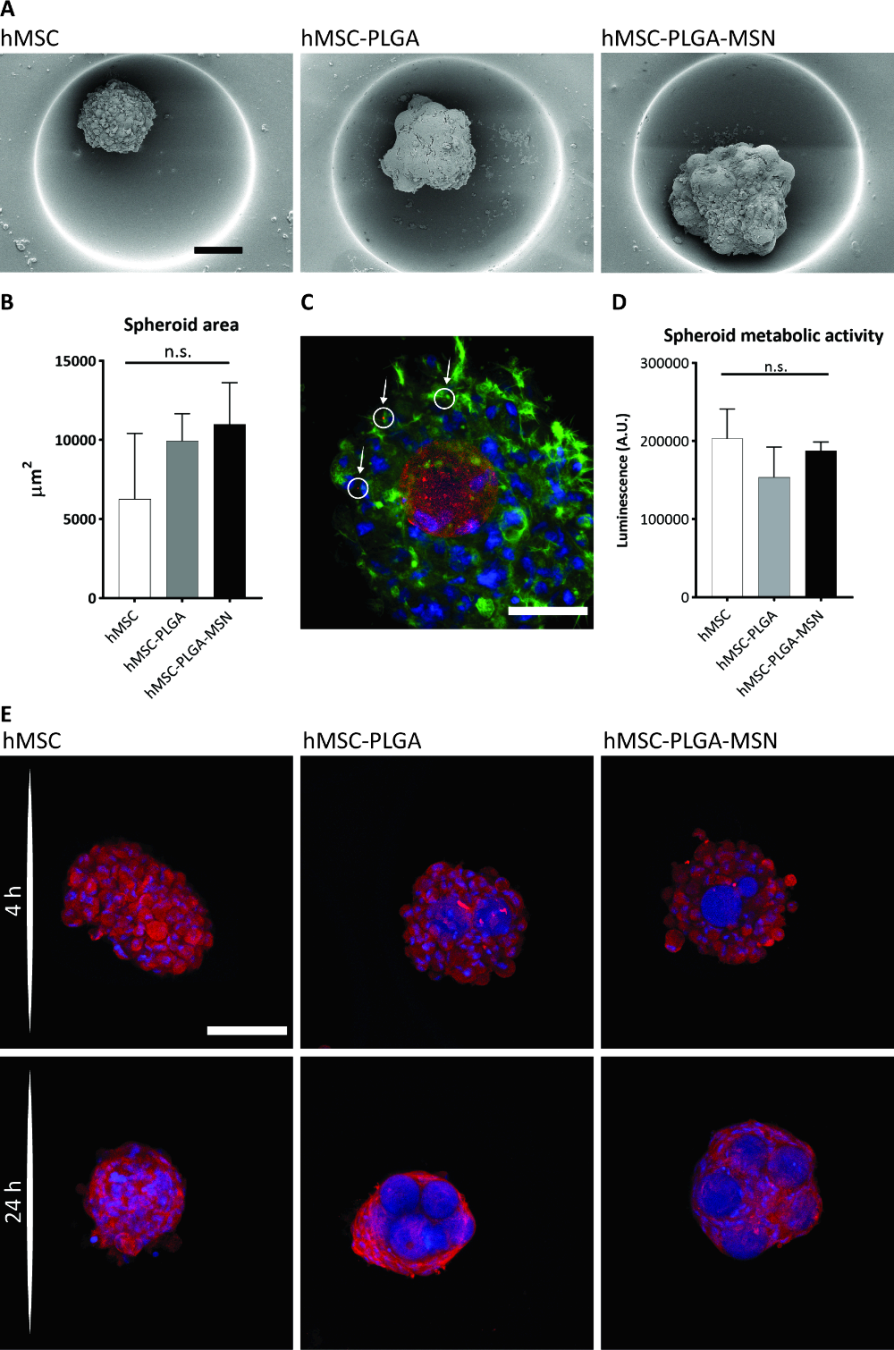
Formation and characterization of hMSC-PLGA-MSN spheroids. (A) SEM images of hMSC and hMSC-(nanofunctionalized) microparticle spheroids after 3 days of culture. The spheroids are formed with about 3 μg of either PLGA (hMSC-PLGA) or MSN-coated PLGA (hMSC-PLGA-MSN). Spheroids formed from hMSCs only (hMSC) served as a control. Scale bar represents 100 μm and applies for all images of the subfigure. (B) Quantification of the projected area of hMSC-nanofunctionalized microparticle spheroids after 3 days of culture by quantitative analysis of confocal fluorescence microscopy images in CellProfiler (*n* = 3 spheroids). ‘n.s.’ stands for ‘not statistically significant’. Statistical method used is one-way ANOVA with Tukey’s post hoc test for multiple comparisons. (C) Maximum projection of a confocal microscopy image of a hMSC-PLGA-MSN spheroid after 24 h of culture. Cells are stained with phalloidin (in green) and DAPI (in blue) to label cytoskeletal F-actin and cell nuclei, respectively. MSNs are labeled with an ATTO647N-Maleimide (in red). Scale bar represents 50 μm. (D) Quantification of cell metabolic activity in spheroids after 3 days of culture by using the CellTiterGlo 3D Assay (*n* = 3). ‘A.U.’ stands for ‘arbitrary units’ and ‘n.s.’ stands for ‘not statistically significant’. The statistical method used is one-way ANOVA with Tukey’s post hoc test for multiple comparisons. (E) Maximum projection of confocal microscopy images of spheroids after (top row) 4 and (bottom row) 24 h. Cells are stained with an antivinculin antibody (in red) and DAPI (in blue) to label focal adhesion points and cell nuclei, respectively. Scale bar represents 100 μm and applies for all images of the subfigure.

We assessed the spheroid formation and evaluated the spheroid morphology over the culture time in the presence of PLGA and PLGA-MSN microparticles. The spheroids successfully formed when PLGA and PLGA-MSN microparticles were included in the microwells, similar to hMSC spheroids grown in the absence of materials (Figure 4A). HMSC-PLGA and hMSC-PLGA-MSN spheroids appeared similar in size and texture after 3 days in culture, showing complete inclusion of the biomaterials within the spheroid volume. Quantification of the spheroid (projection) area was performed via an image analysis. There was no significant difference between the average areas of the hMSC-only, hMSC-PLGA, and hMSC-PLGA-MSN spheroids after 3 days in culture (Figure 4B). This could be due to the small amount of microparticles added in the spheroids (3 μg, equal to only approximately 2.5 nL). To better characterize spheroid compaction over time for the different spheroid conditions, MSNs were labeled with an ATTO647N-Maleimide dye to allow visualization with a fluorescence microscope. The spheroid formation within the microwells started immediately, and cells and biomaterials visibly aggregated within the first 12 h at the latest (Video S1A–C). After 12 h, their compaction slowed down to eventually stabilize, and they maintained the same size for the rest of the observation. The presence of biomaterial microparticles seemed to speed up the aggregation process. Both hMSC-PLGA and hMSC-PLGA-MSN spheroids formed within the first 4 to 6 h, while hMSC spheroids aggregated in the first 8 h. Some concurring factors may explain this observation. The aggregation of the hMSC-PLGA and hMSC-PLGA-MSN spheroids might be supported by the inclusion of PLGA, the negative charge of which is reported to be firmly bound to cells in aggregates.^51^ MSNs are also known to enhance cell attachment and proliferation in hydrogels.^52^ Similar processes might have occurred within the spheroids as well.

Next, MSN release from the microparticles and transport of the loose MSNs within the spheroid microenvironment was investigated. A high magnification image of hMSC-PLGA-MSN spheroids after 48 h of culture revealed that the MSNs remained evenly coated on the PLGA microparticles (Figure 4C). Minimal MSN release from the PLGA surface to the intercellular space of the spheroid could be observed (indicated by the white arrows in the white circles). This indicated that the MSN coating on the PLGA microparticles was stable for up to 48 h when the nanofunctionalized microparticles were combined into spheroids.

### 3.5 HMSCs Show Metabolic Activity in hMSC-Nanofunctionalized Microparticle Spheroids

Material-driven effects on cell viability do not only depend on the biomaterial chemistry but also on biomaterial size and shape and differs between 2D and 3D settings.^53^ The metabolic activity of the hMSCs within hMSC-nanofunctionalized microparticle spheroids was evaluated after 3 days in culture in basic medium by quantifying the total ATP production, a biomarker correlated to metabolically active cells.^54^ As shown in Figure 4D, similar ATP production was observed in hMSC, hMSC-PLGA, and hMSC-PLGA-MSN spheroids, suggesting that all spheroid conditions contained similar numbers of metabolically active cells. Indeed, both PLGA microparticles^55^ and MSNs are known to be biocompatible,^39^ supporting cell attachment and metabolic activity.

### 3.6 HMSC-PLGA Spheroids Present Similar Vinculin Expression with or without the Inclusion of MSNs

The assessment of focal adhesion formation when cells get in contact with a substrate is one of the first and most important aspects to consider when investigating the cytocompatibility of a substrate.^56^ Vinculin is a protein involved in several cellular processes including the formation of focal adhesions.^57^ There, it contributes to the formation of adhesion plaques in the cytoplasmic domain when cell adhesion occurs.^56^ Here, the immunolabeling of vinculin was performed to better understand the biological mechanisms underneath the spheroid formation in the presence of PLGA and PLGA-MSN microparticles. The expression of vinculin for the hMSC-nanofunctionalized microparticle spheroids was qualitatively evaluated via image analysis of immunofluorescence images after 4 and 24 h of culture (Figure 4E). The expression of vinculin was comparable for the hMSC, hMSC-PLGA, and hMSC-PLGA-MSN spheroids. In all conditions, vinculin expression was abundant and homogeneously distributed over the spheroid. After 4 h in culture, the vinculin expression appeared quite dispersed and disorganized, resulting in a uniform fluorescent stain of the whole cell area. After 24 h, though, the expression of vinculin exhibited a more organized structure, indicating maturing focal adhesions.^58^

The inclusion of PLGA and PLGA-MSN microparticles did not substantially change vinculin expression. The hybrid cell-(nanofunctionalized) microparticle spheroid formation pattern was comparable to that of hMSC spheroids without biomaterials. Hence, we can assume that the inclusion of microparticles, both PLGA and PLGA-MSN ones, did not affect the intrinsic cell organization, allowing for spheroid self-organization within the microwells. This was considered an essential result for further analysis, as an uncompromised spheroid formation was necessary for a meaningful further investigation of the potential of the MSNs as nanocarriers of nutrients.

### 3.7 Tunable Release of Fluorescently Labeled Glucose from MSNs and PLGA-MSNs Was Monitored by Changing the Loading Concentration

MSNs are widely used as delivery vehicles for different types of cargos such as anticancer drugs and genes.^59,60^ Here, we explored the capacity of MSNs as nutrient reservoirs. Glucose was selected as our cargo to improve the viability of hMSC spheroids by releasing this molecule locally within the spheroids. We first used a fluorescently labeled deoxyglucose analogue, Glu_F_, to perform the release studies. To show the influence of the Glu_F_ loading concentration on the release behavior of MSNs, different concentrations of Glu_F_ (low, medium, and high concentrations of Glu_F_ of 1, 10, and 100 mM, respectively) were loaded into the MSNs and the release of Glu_F_ was monitored by a fluorescence spectrometer for 24 h. As expected, the results revealed that the highest Glu_F_ release was observed when the MSNs were loaded with a high concentration of Glu_F_ followed by medium and low Glu_F_ loading (Figure S6).

**Figure 5:**
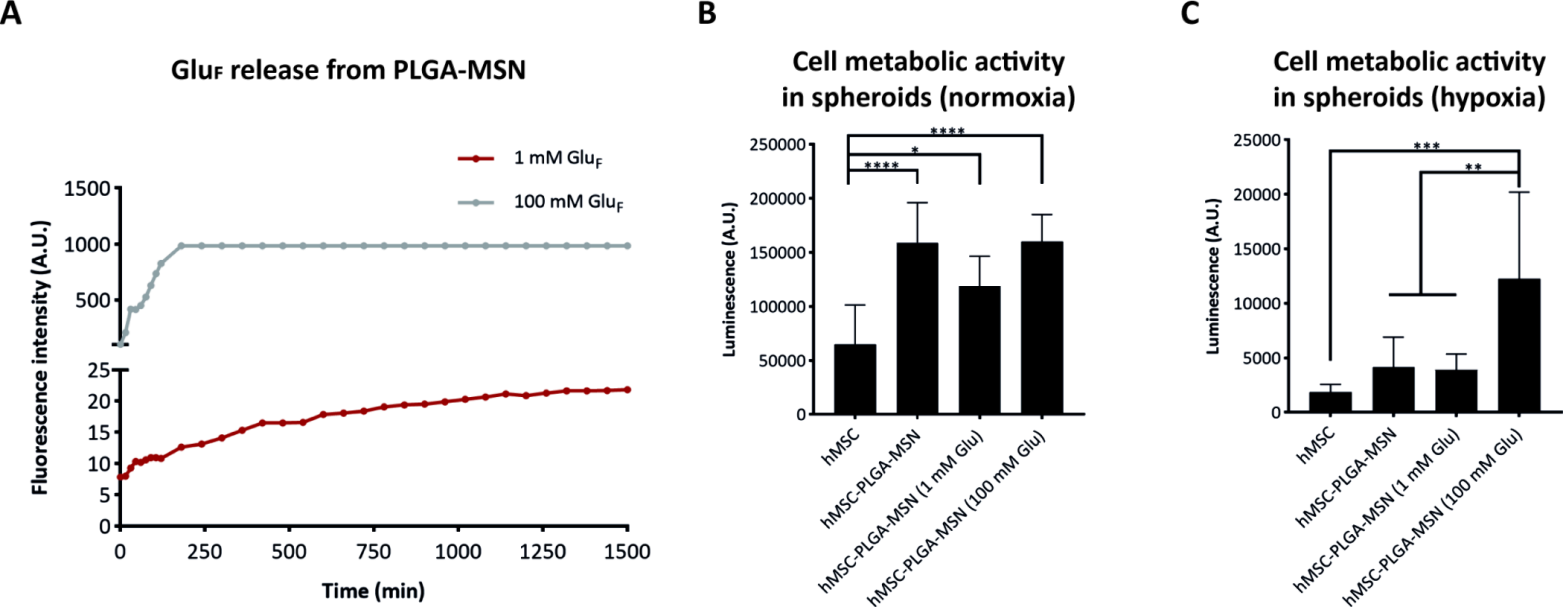
Glu_F_ release from PLGA-MSNs and glucose effect on cell viability in hMSC-nanofunctionalized microparticle spheroids. (A) Glu_F_ release from PLGA-MSNs for 1 and 100 Mm Glu_F_ loading. Quantification of cell metabolic activity in spheroids in (B) normoxia and (C) hypoxia (5% pO_2_) conditions after 1 day of culture in DPBS (*n* = 3) by using the CellTiterGlo Assay. ‘*’, ‘**’, ‘***’, and ‘****’ represent p values smaller than 0.05, 0.01, 0.001, and 0.0001, respectively. ‘A.U.’ stands for ‘arbitrary units’. Statistical method used is one-way ANOVA with Tukey’s post hoc test for multiple comparisons.

Afterward, we examined the release of Glu_F_ from PLGA-MSN microparticles loaded with 1 mM Glu_F_ (PLGA-MSN (1 mM Glu_F_)) and with 100 mM Glu_F_ (PLGA-MSN (100 mM Glu_F_)) during 24 h (Figure 5A). These concentrations were chosen to monitor drastic changes in the release behavior of MSNs as a function of glucose loading concentration. PLGA-MSN (1 mM Glu_F_) exhibited steady release over the 24 h incubation period. The concentration of released Glu_F_ was calculated based on a calibration curve (Figure S7) and after 24 h was approximately 0.1 μM for PLGA-MSN (1 mM Glu_F_). Glucose release from PLGA-MSN loaded with 100 mM Glu_F_ was expectedly higher than that from the PLGA–MSNs with 10 mM Glu_F_ loading. However, the release of glucose in this condition could reliably only be measured in the first 3 h. After this time, due to the high amount of released glucose, the fluorescence signal in the sample reached a level above the detection limit of the spectrophotometer. However, it could be observed that the concentration of the released glucose from the MSN coatings in PLGA-MSN (100 mM Glu_F_) had reached 5 μM only after 3–4 h. It should be noted that the final concentration of released Glu_F_ for PLGA-MSN (100 mM Glu_F_) might be different from the real value due to the detection limit of the equipment as mentioned earlier. Previously, Schop et al. calculated a glucose consumption of 9.2 pmol/cell/day for hMSCs in normoxic conditions.^61^ In our study, cell-nanofunctionalized microparticle spheroids are formed by nearly 5000 cells. Based on these assumptions, the theoretical glucose consumption per hMSC spheroid is approximately 0.046 μmol/day. Considering that each microwell array contains 30 microwells, of which each of them hosts one spheroid, this adds up to a maximal theoretical glucose consumption of 1.38 μmol/day per complete microwell array. The two calculated glucose release concentrations in our system (0.1 and 5 μM) provide glucose amounts of 0.02 nmol (after 24 h) and 1 nmol (after 3–4 h), respectively, which are lower than this maximal theoretical value, suggesting that higher glucose loading may be even more beneficial for cell-nanofunctionalized microparticle spheroids. Overall, our results showed that the amount of released glucose (Glu_F_) can be modulated by changing its initial MSN loading concentration.

### 3.8 Glucose Loading of the MSNs Positively Affects Metabolic Activity of hMSCs in hMSC-Nanofunctionalized Microparticle Spheroids in Both Normoxia and Hypoxia

As a next step, we evaluated the effect of the released glucose from the PLGA-MSN biomaterials on the metabolic activity of hMSCs by quantifying total ATP per sample. For this experiment, the cell culture was performed in both normoxic and hypoxic (pO_2_ = 5%) conditions in DPBS after 1 (Figure 5B,C), 3, and 7 days (Figure S8A,B). The choice of culturing the spheroids in DPBS was made to investigate solely the effect of the glucose-loaded MSNs on metabolic activity, without the impact of glucose in standard culture medium formulations, which is 5.5 mM in DMEM. Also, it prevented altered results from a combined interplay of glucose with other culture medium components on the hMSC metabolism, such as sodium pyruvate.^33^ At day 1 and under normoxic conditions (Figure 5B), the cell metabolic activity was notably improved by the presence of the nanofunctionalized microparticles. The presence of PLGA-MSN and PLGA-MSN loaded with 1 mM (hMSC-PLGA-MSN (1 mM Glu)) and 100 mM glucose (hMSC-PLGA-MSN (100 mM Glu)) significantly improved the hMSC metabolic activity in spheroids when compared with the values for pure hMSC spheroids. The effect of MSN-mediated glucose release on the metabolic activity of hMSCs was more pronounced under hypoxic conditions (Figure 5C). In the harsh condition of hypoxia, lower ATP production was recorded for all the conditions. However, cell metabolic activity for the condition hMSC-PLGA-MSN (100 mM Glu) was significantly higher than the other conditions (Figure 5C). In normoxia, the metabolic activity of the cells within the spheroids was significantly lower after 3 and 7 days in culture compared to what was observed after 1 day (Figure S8A), likely because all glucose was released within the first 24 h (Figure 5A). In hypoxia, except for the condition hMSC-PLGA-MSN (100 mM Glu) at day 1, the other conditions and time points showed overall low ATP production and no statistically relevant differences (Figure S8B), indicating no positive effect of the nanofunctionalized microparticle-based glucose delivery system on the metabolic activity of the cells in spheroids at time points later than day 1.

Overall, we showed that the proposed nanofunctionalized microparticle-based glucose delivery system improved the metabolic activity of hMSCs in the spheroids under both normoxic and hypoxic conditions in short-term cultures. As previously reported, glucose diffusion through the spheroid volume plays a crucial role in spheroid homeostasis and the maintenance of its 3D architecture.^62^ HMSC glycolysis is described as the prevailing metabolic activity leading to cell proliferation and chondrogenic differentiation in 2D^63^ and, overall, glucose is described as the most important metabolic substrate to influence hMSC differentiation.^63^ In 3D culture in hMSC spheroids, insights on glucose diffusion have also been given, highlighting a correlation between the spheroid size and the glucose consumption.^64^ Therefore, the bigger the spheroid, the lower the glucose consumption may be because of a decreasing ability of glucose to reach the inner spheroid core. In 2D culture, it is narrated that in severe hypoxia conditions (pO_2_ < 1.5%), hMSCs can survive in culture as long as being fed with glucose.^65^ Moreover, studies on hMSCs in near-anoxia conditions (pO_2_ = 0.1%) reported how glucose and glycolysis are the only energy source and metabolic pathways, respectively, to ensure cell survival in these extreme conditions.^33^ In this context, our findings are in line with the available literature. However, the 3D architecture of spheroids should also be considered as a concurrent factor, as it may amplify the hypoxic effect on cell metabolic activity even when glucose is provided.

### 3.9 Glucose Release from Glucose-Loaded PLGA-MSN Microparticles Reduces Cell Death in Cell Spheroids in Hypoxia

**Figure 6:**
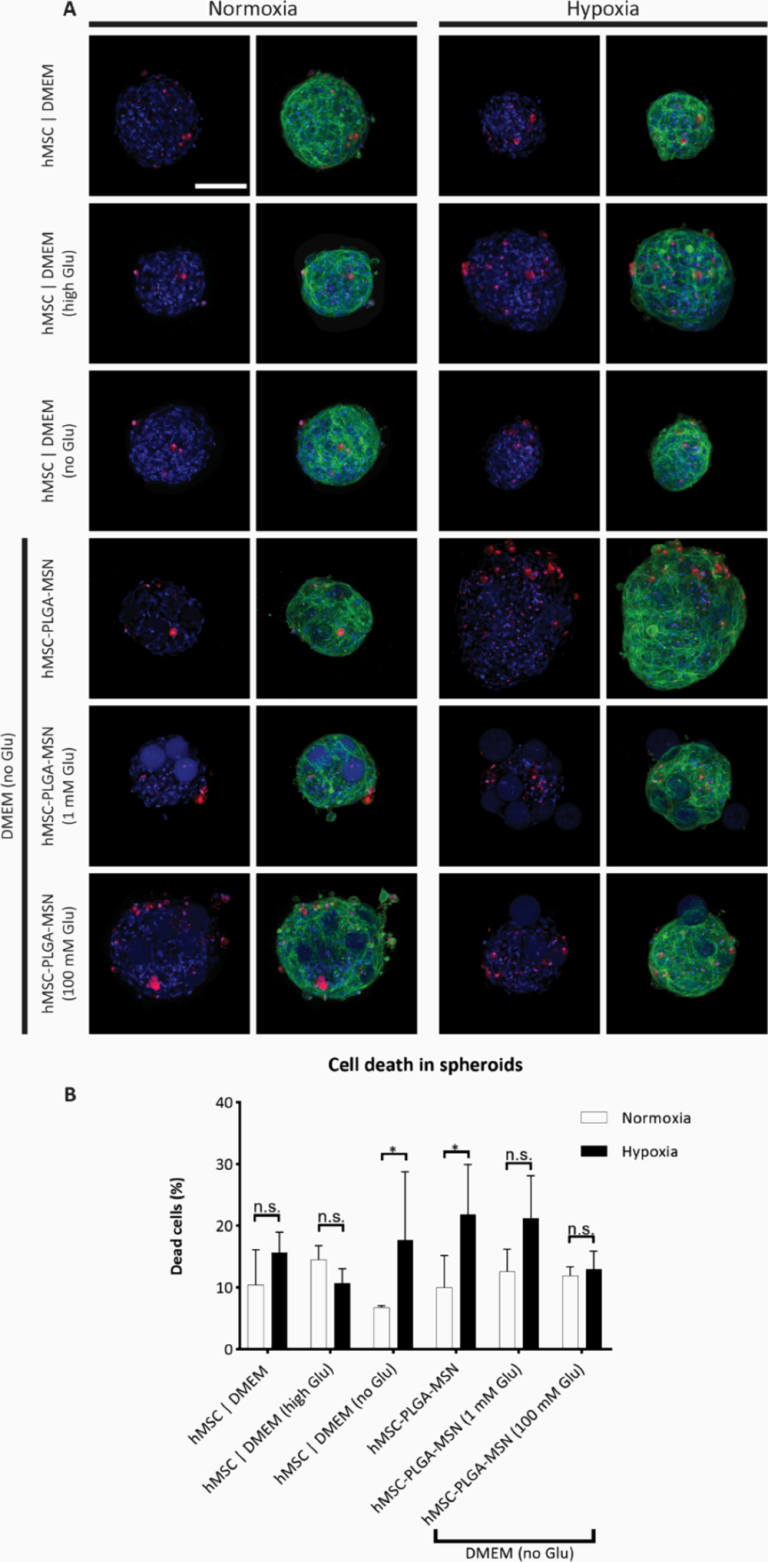
Glucose release effect on cell death in hMSC-nanofunctionalized microparticle spheroids. (A) Maximum projection of confocal microscopy images of spheroids after 24 h of culture. Cells are stained with Fixable Far Red Dead Cell Stain (in red), DAPI (in blue), and phalloidin additionally (in each case right column of the two double columns, in green) to label dead cells, cell nuclei, and F-actin, respectively. Scale bar represents 100 μm and applies for all images of the subfigure. (B) Quantification of cell viability in spheroids in normoxia and hypoxia conditions (5% pO_2_) after 24 h of culture by quantitative analysis of confocal fluorescence microscopy images in CellProfiler (*n* = 4 spheroids). ‘*’ represents *p* values smaller than 0.05 and ‘n.s.’ stands for ‘not statistically significant’. Statistical method used is two-way ANOVA with Tukey’s post hoc test for multiple comparisons.

Cell death within cell-nanofunctionalized microparticle spheroids where the MSNs were loaded with different amounts of glucose was evaluated after 1 (Figure 6A,B) and 7 days of culture in a glucose-free DMEM (DMEM (no Glu)) under normoxic and hypoxic conditions (Figure S9). HMSC spheroids in basic medium, containing 5.5 mM glucose, as well as hMSC spheroids cultured in high glucose (25 mM glucose) DMEM served as positive controls. Spheroids formed in all media and the presence of all biomaterials, regardless of the oxygen partial pressure. Cell death was quantified via image analysis of the colocalization loci of dead cells and cell nuclei, which were previously fluorescently labeled and expressed as a percentage of the total number of cells (i.e., total cell nuclei number). A first qualitative observation shows that the majority of the cells within the spheroids in normoxic conditions were alive after 1 day of culture, with very small amounts of dead cells for all of the designed conditions (Figure 6A; “normoxia”, left column). At the same time point, a mostly higher amount of dead cells could be identified for spheroids cultured in a hypoxic environment (Figure 6A; “hypoxia”, right column). This observation was particularly evident for the conditions hMSC-PLGA-MSN and hMSC-PLGA-MSN (1 mM Glu).

The quantification of dead cells through analysis of the confocal fluorescence images confirmed the qualitative observations described above (Figure 6B). After 1 day in culture, comparatively low levels of cell death were observed in normoxic conditions. However, in hypoxia, hMSC spheroids cultured in DMEM and high glucose DMEM (hMSC | DMEM and hMSC | DMEM (high Glu)) and hMSC-nanofunctionalized microparticle spheroids with 100 mM Glu (hMSC-PLGA-MSN (100 mM)) showed lower values of cell death in comparison to conditions without glucose. The presence of glucose released from MSNs showed a clear positive effect on maintaining cell viability in spheroids in the hypoxic environment similar to normoxic conditions, contrasting the detrimental effect of hypoxia on stromal cell survival.^66^ Evidence of this occurrence was already reported in the literature for murine bone marrow^67^ and sheep-derived MSCs in 2D cultures,^65^ but the reproduction of this phenomenon in 3D in spheroids was not reported so far. It is worth mentioning that here the glucose loaded in MSNs was estimated to be thousands of times less than the amount diluted in basic DMEM. This suggests that the biomaterial-based glucose delivery system may be more efficient in maintaining and influencing cell viability, in comparison with the diffusion mechanism from the medium surrounding the spheroids. As a consequence of these findings, a possible advantage of the nanofunctionalized microparticle-based glucose delivery system may be its local action within the spheroid volume.

Similar to what was observed for the spheroids in DPBS, the beneficial effect of the glucose-releasing nanofunctionalized microparticles on cell viability was not sustained over time. Cell death, as described earlier for DPBS, was quantified after 7 days in the cell culture medium as well. In this instance, no media exchange was performed to not alter the effect of the glucose-releasing biomaterials. No statistically significant differences were revealed between hypoxic and normoxic culture conditions (Figure S9). Importantly, equal amounts of cell death were obtained for all conditions, including those for the spheroids in high-glucose DMEM in normoxia. This indicates that cell death was independent of the nanofunctionalized microparticle delivery system and occurred naturally as a consequence of the experimental design.

**Figure 7:**
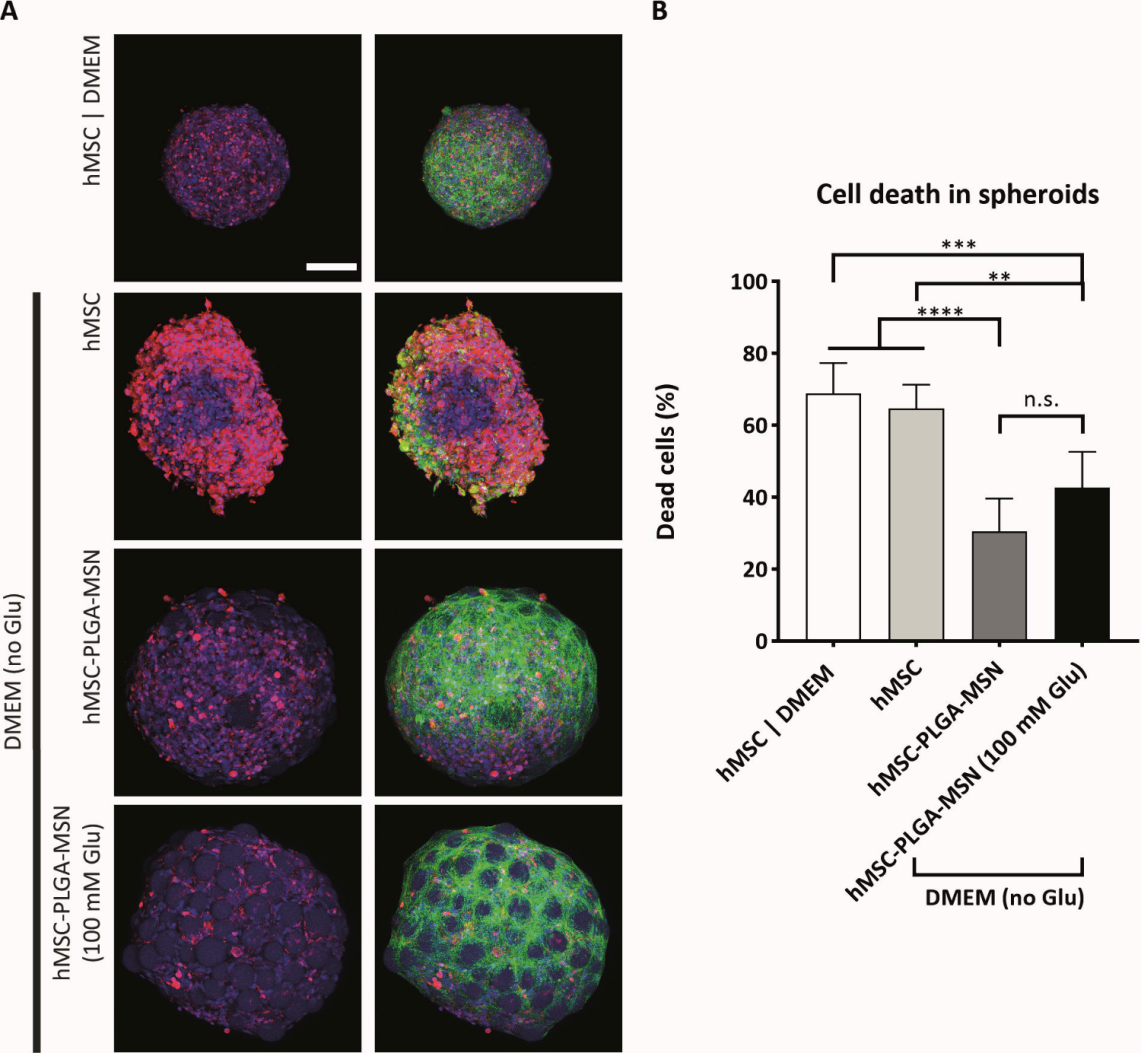
Glucose release effect on cell death in large hMSC-nanofunctionalized microparticle spheroids. (A) Maximum projection of confocal microscopy images of larger spheroids after 3 days of culture in normoxic condition. Cells are stained with Fixable Far Red Dead Cell Stain (in red), DAPI (in blue), and phalloidin (in each case right column of the two double columns, in green) to label dead cells, cell nuclei, and F-actin, respectively. Scale bar represents 100 μm and applies for all images of the subfigure. (B) Quantification of cell death in spheroids in normoxia after 3 days of culture by quantitative analysis of confocal fluorescence microscopy images in CellProfiler (*n* = 6 spheroids). ‘**’, ‘***’, and ‘****’ represent *p* values smaller than 0.05, 0.01, and 0.001, respectively. ‘n.s.’ stands for ‘not statistically significant’. Statistical method used is one-way ANOVA with Tukey’s post hoc test for multiple comparisons.

To investigate the effects of the nanofunctionalized microparticles on reducing the necrotic regions in spheroids, we formed larger spheroids with two times higher amounts of cells and microparticles compared to the previous experiments. It is known that, even in a normoxic environment, increasing the spheroid size can lead to the formation of a layered structure in spheroids, consisting of a necrotic inner core, a middle layer of quiescent cells, and an outer layer of proliferating cells.^68^ We observed that the control spheroids showed distinct dark cores after 3 days, potentially indicating the formation of necrotic cores. The visualization and quantification of dead cells showed that the control large spheroids formed in normal and glucose-free DMEM had substantially higher cell death levels (over 60%, Figure 7A,B) as compared to their smaller counterparts with 10–20% cell death (Figure 6A,B), indicating the formation of necrotic regions under these conditions. Of interest, the cell death was significantly reduced in the spheroids containing nanofunctionalized microparticles (even without glucose loading) in glucose-free medium as compared to the controls (Figure 7A,B). This means that in large spheroids, the presence of nanofunctionalized microparticles had a more dominant role in reducing necrotic core compared to that of the glucose cargo release in the short term. This may be due to the decreased compaction in cell-biomaterial spheroids,^3^ which may facilitate the exposure of the cells to the oxygen-rich medium and other nutrients. In addition, MSN coatings, known as biocompatible biomaterials, may have increased the cell viability in hMSC spheroids.

## 4 Conclusions and Outlook

In this study, we propose a new nanofunctionalized microparticle-based delivery system that might serve as a versatile tool for the local delivery of nutrients and bioactive factors in cell spheroids. We exploited the highly porous structure of MSNs as nanoreservoirs for loading small molecules and introduced the loaded MSNs as a coating onto the PLGA microparticles. These were coseeded with hMSCs to form cell-nanofunctionalized microparticle spheroids. We coated the surface of PLGA microparticles homogeneously with MSNs based on electrostatic interactions and evaluated the stability of the MSN coating on the microparticles in cell culture conditions. Then, the basic biological performance of the cell-nanofunctionalized microparticle spheroids was investigated in terms of the morphological appearance, viability, and focal adhesion expression. This investigation proved that the designed nanofunctionalized microparticles were biocompatible and did not hinder the aggregation of hMSCs, which was similar to the cell-only spheroids. As a proof of concept, we loaded fluorescently labeled glucose into MSNs with different loading concentrations and monitored their release behavior. For cell culture studies, we successfully loaded glucose into the MSNs and investigated its effect on spheroids’ viability, expressed as ATP production and cell death. Overall, our results showed the integration of glucose-releasing nanofunctionalized microparticles within spheroids, which resulted in the improved viability of the cells, particularly in hypoxic conditions during short periods of culture. The viability of the cells within spheroids provided with glucose through the nanofunctionalized microparticles was comparable with what was observed in conditions of normal glucose diffusion from the medium, despite smaller amount of glucose loaded into the MSNs. This highlights the role of the nanofunctionalized microparticle-based delivery system as a system of more confined and local glucose supply to the spheroids and the cells therein to obtain a more homogeneous distribution of glucose throughout the spheroid volume. In addition to effective release of glucose in spheroids, our approach was advantageous for reducing the inner necrotic core of spheroids, which was observed when a higher number of cells were used to produce larger spheroids.

In future applications, our work may present a versatile delivery system for different types of small molecules and soluble compounds. In particular, this system may be suitable to the prompt release of therapeutics that require immediate action, such as antibiotics^69^ and antimicrobials,^70^ by exploiting its local effect, thus maximizing the effective dosage. Moreover, future efforts may focus on the optimization of biomaterial-based delivery systems with a sustained release profile over time, for example, by exploring a wider range of loaded compound concentrations or by introducing different types of coatings, for example, from polymers or lipids, on the MSN surface to slow down the cargo release to the spheroid/3D cell culture constructs. Stimuli-responsive biomaterial coatings, which respond to pH or temperature triggers, may add an extra degree of control to the development of more precise and tunable drug/soluble biofactor delivery systems.

## Data Availability

Data will be made available on request.

## Supporting Information Available

Additional data on MSN coating stability on PLGA microparticles, visualization of bare PE and PMMA microparticles before oxygen plasma treatment, the stability of MSN-coated PE and PMMA microparticles, Glu_F_ release profile from MSNs in suspension, calibration curve of Glu_F_, and glucose release effect on cell viability and death in spheroids (PDF)
Videos of aggregation of hMSC, hMSC-PLGA, and hMSC-PLGA-MSN spheroids over time (MP4, MP4, and MP4)
