# Supplementary material for: Nanofunctionalized Microparticles for Glucose Delivery in Three-Dimensional Cell Assemblies
Source: ACS Appl Mater Interfaces. 2024 Apr 2;16(14):17347–60. doi: 10.1021/acsami.4c02321 (PMC11009907; doi:10.1021/acsami.4c02321)
Supplement: Supplementary file 1 — am4c02321_si_001.pdf [file am4c02321_si_001.pdf]

**Supporting information**

# Nanofunctionalized Microparticles for Glucose Delivery in Three-Dimensional Cell Assemblies

Maria G. Fois[1#], Aygul Zengin[1#], Ke Song[1], Stefan Giselbrecht[1], Pamela Habibović[1], Roman K. Truckenmüller[1], Sabine van Rijt[1*##] and Zeinab N. Tahmasebi Birgani[1*##]

[1]Department of Instructive Biomaterials Engineering, MERLN Institute for Technology-Inspired Regenerative Medicine, Maastricht University, P.O. Box 616, 6200 MD Maastricht, The Netherlands

#, ## authors contributed equally to this work

* Corresponding authors:
Sabine van Rijt, PhD; s.vanrijt@maastrichtuniversity.nl
Zeinab N. Tahmasebi Birgani, PhD; z.tahmasebibirgani@maastrichtuniversity.nl

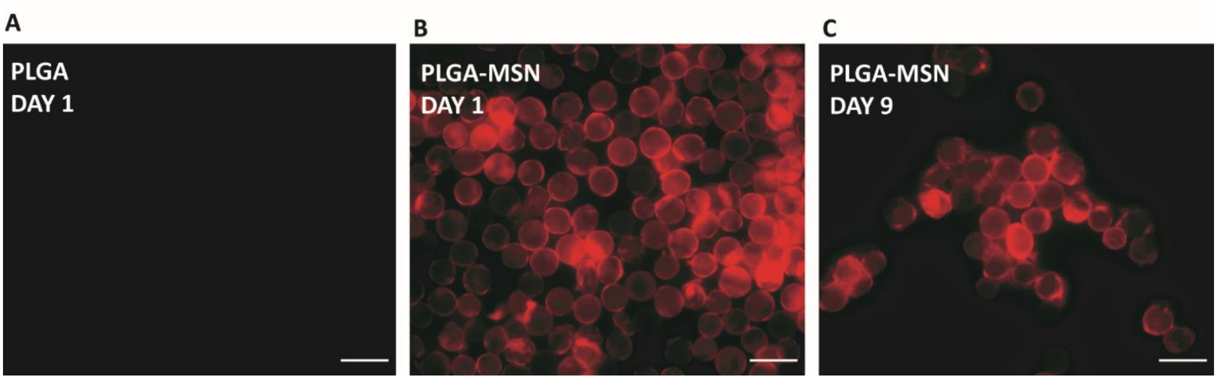


**Figure S1.** *MSN coating stability on PLGA microparticles over time.* Fluorescence images of coating of ATTO647N-Maleimide core-labeled MSNs on PLGA microparticles. (A) Bare PLGA microparticles after 1 day incubation in Milli-Q water at RT. (B) PLGA-MSN after 1 day incubation in Milli-Q water at RT. (C) PLGA-MSN after 9 days incubation in Milli-Q water at RT. Scale bar represents 100 μm.

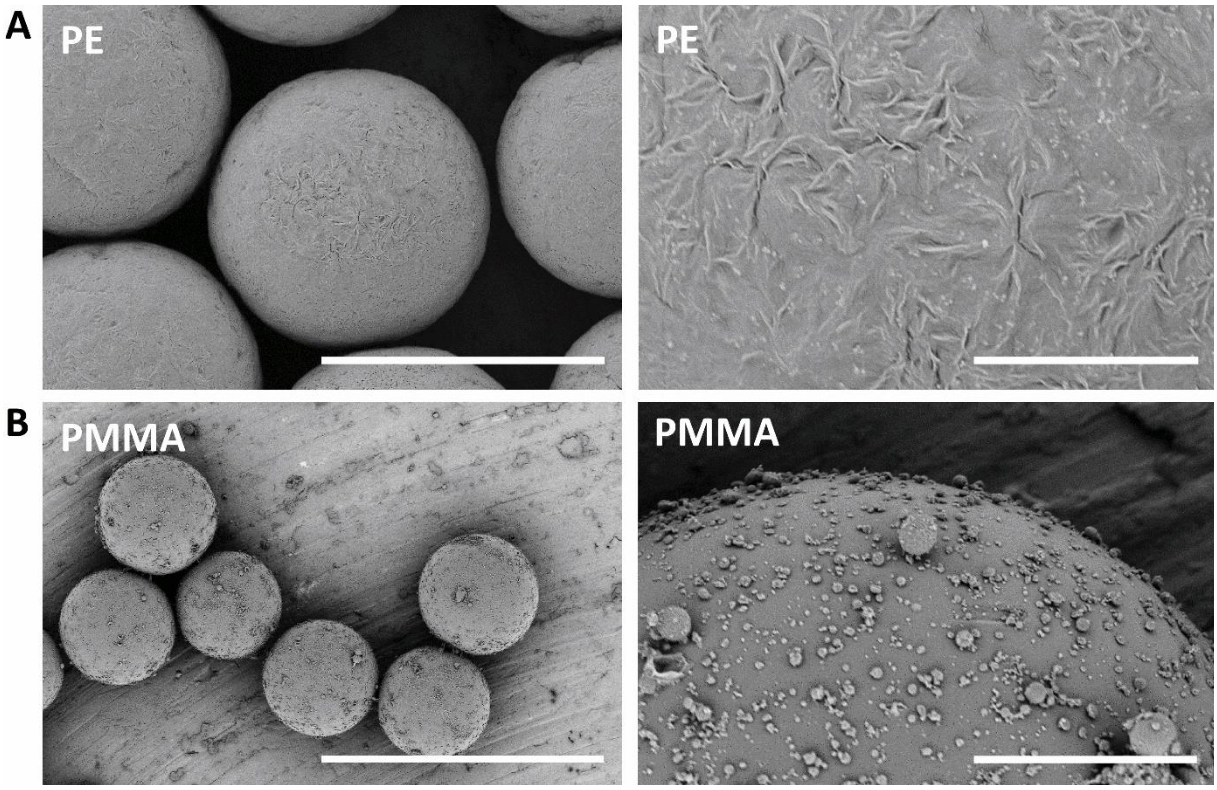


**Figure S2.** *Visualization of bare PE and PMMA microparticles before oxygen plasma treatment.* (A) Bare PE microparticles. Scale bars represent (left image) 50 and (right image) 10 μm. (B) Bare PMMA microparticles. Scale bars represent (left image) 100 and (right image) 10 μm, respectively.

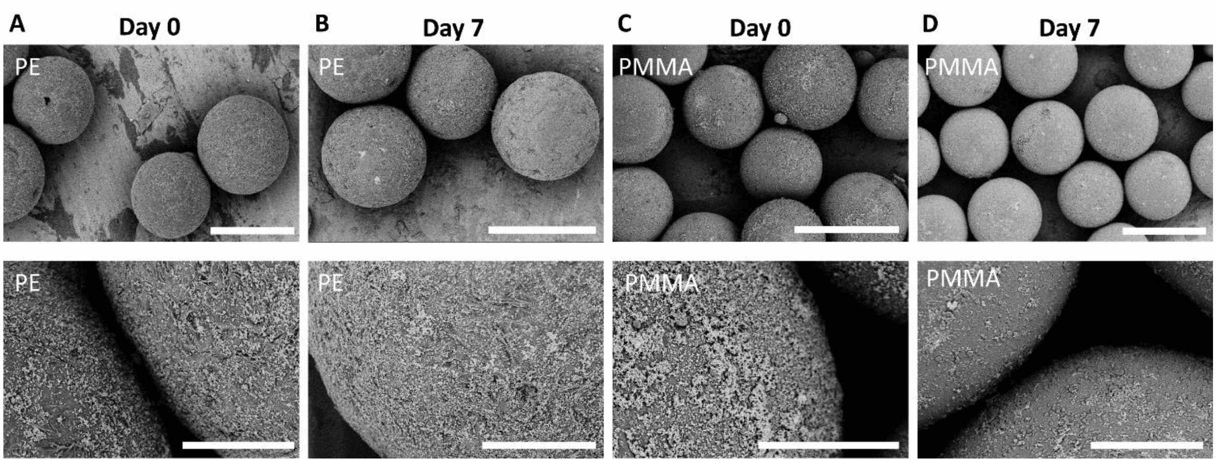


**Figure S3.** *The stability of MSN-coated PE (PE-MSNs) and PMMA (PMMA-MSNs) microparticles in cell culture media during 7 days at 37 °C.* (A) SEM images of PE-MSNs before incubation (Day 0), and (B) after 7 days of incubation. (C) SEM images of PMMA-MSNs before incubation (Day 0), and (D) after 7 days of incubation. Scale bars represent (top images) 50 and (bottom images) 10 µm.

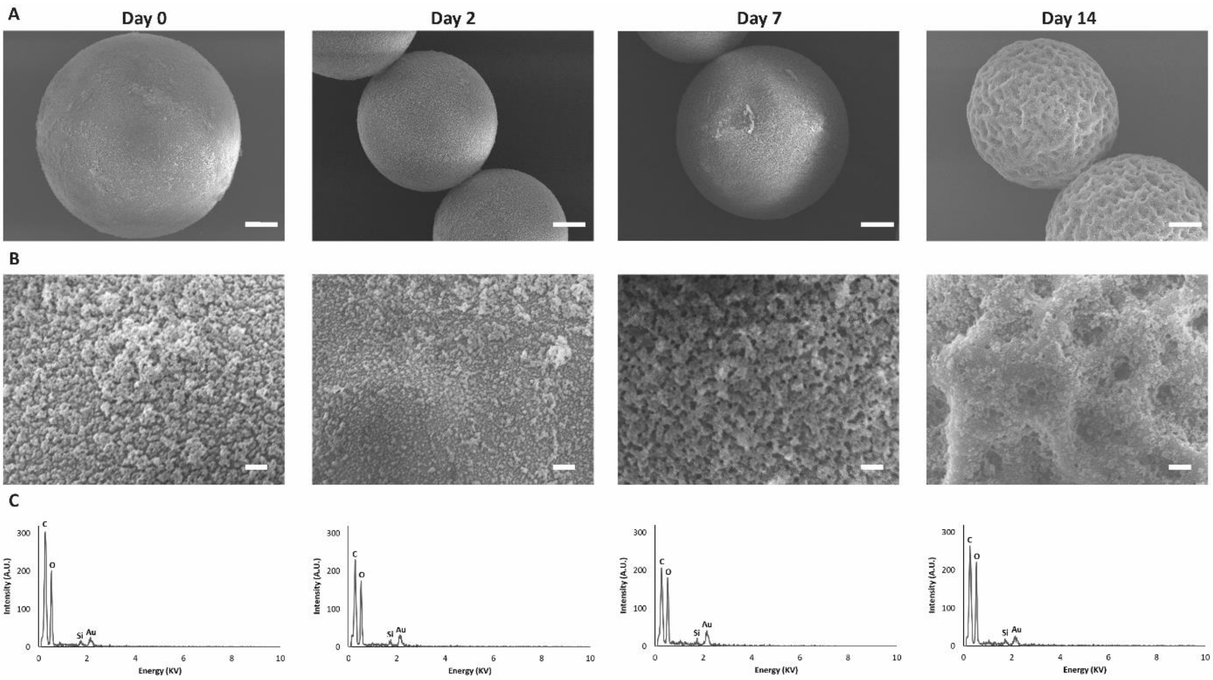


**Figure S4.** *The stability of PLGA-MSN microparticles in cell culture medium containing FBS over the course of 14 days at 37 °C.* SEM images of (A) PLGA-MSN microparticles and (B) close-up images of their surfaces before incubation (day 0), and after 2 days, 7 days, and 14 days of incubation. Scale bars represent 10 µm in A and 2 µm in B. (C) EDS spectra of the surface of PLGA microparticles.

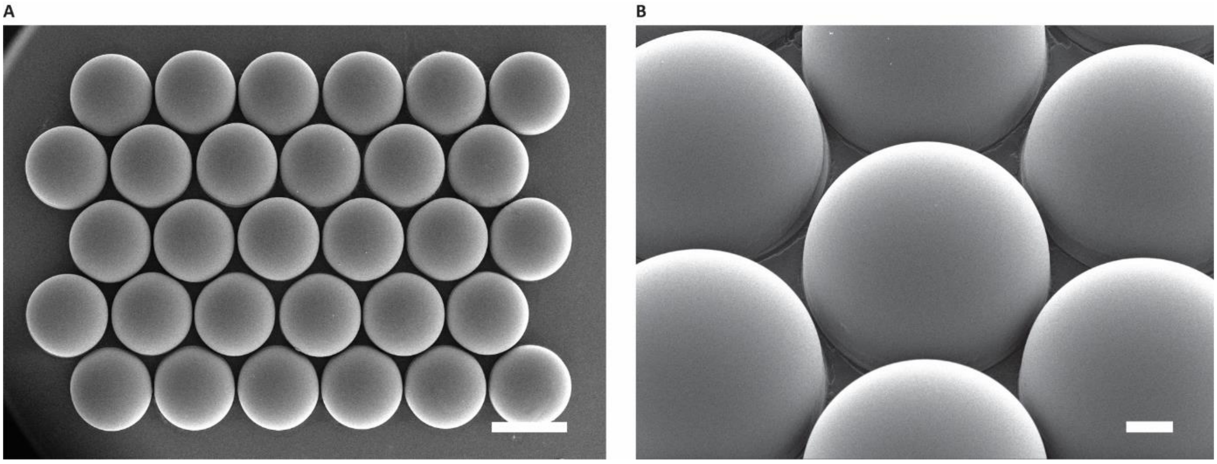


**Figure S5.** *Microwell array appearance.* (A) Bottom view SEM image of the PC 30-microwell array. Scale bar represents 500 µm. (B) Close-up tilted view SEM image of a few microwells. Scale bar represents 50 µm.

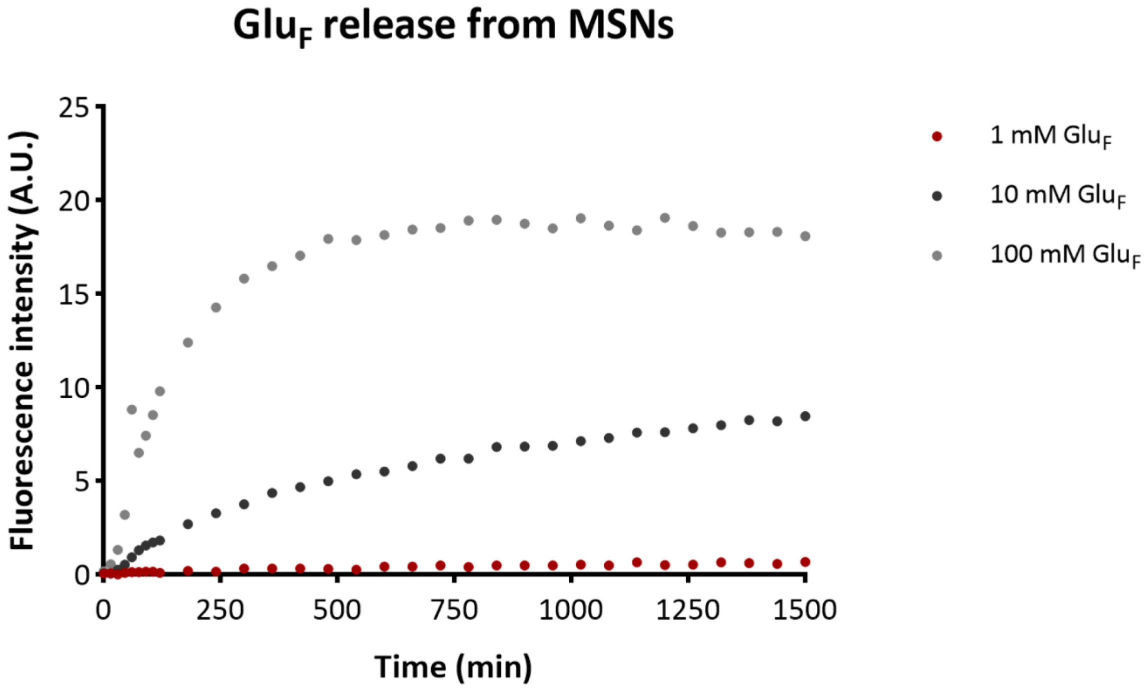


**Figure S6.** *Glu$_F$ release profile from MSNs in suspension*. Glu$_F$ release from MSNs at different loading concentrations of 1 mM, 10 mM, and 100 mM over the course of 24 hours (n=1).

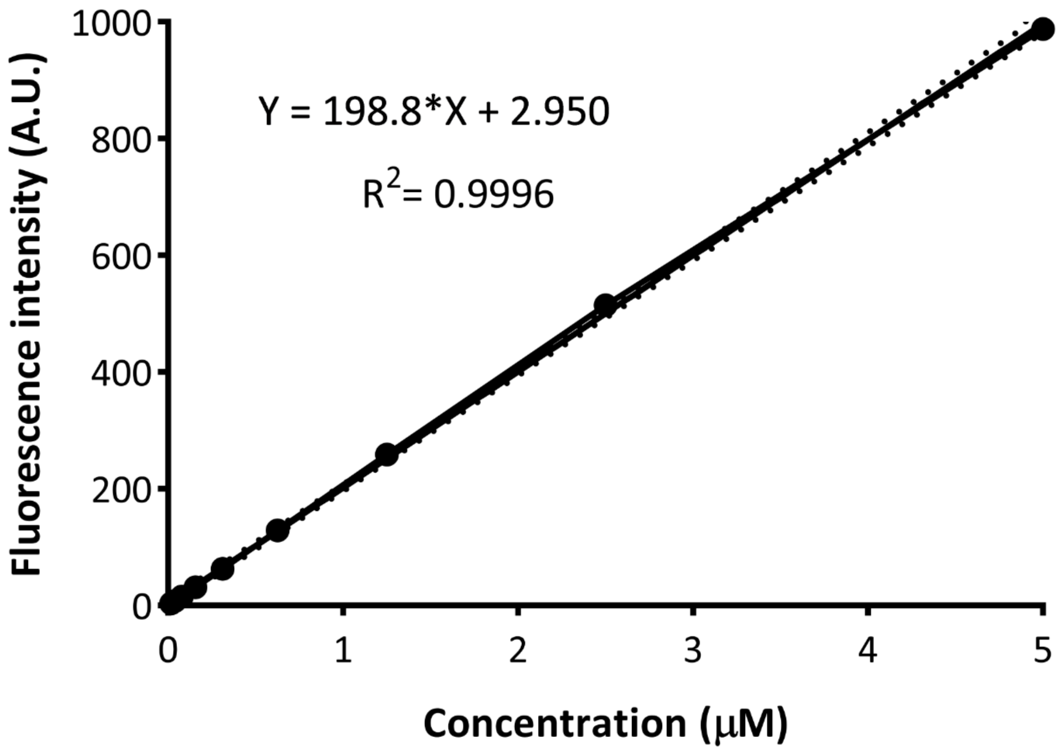


**Figure S7.** *Calibration curve of* $Glu_F$.

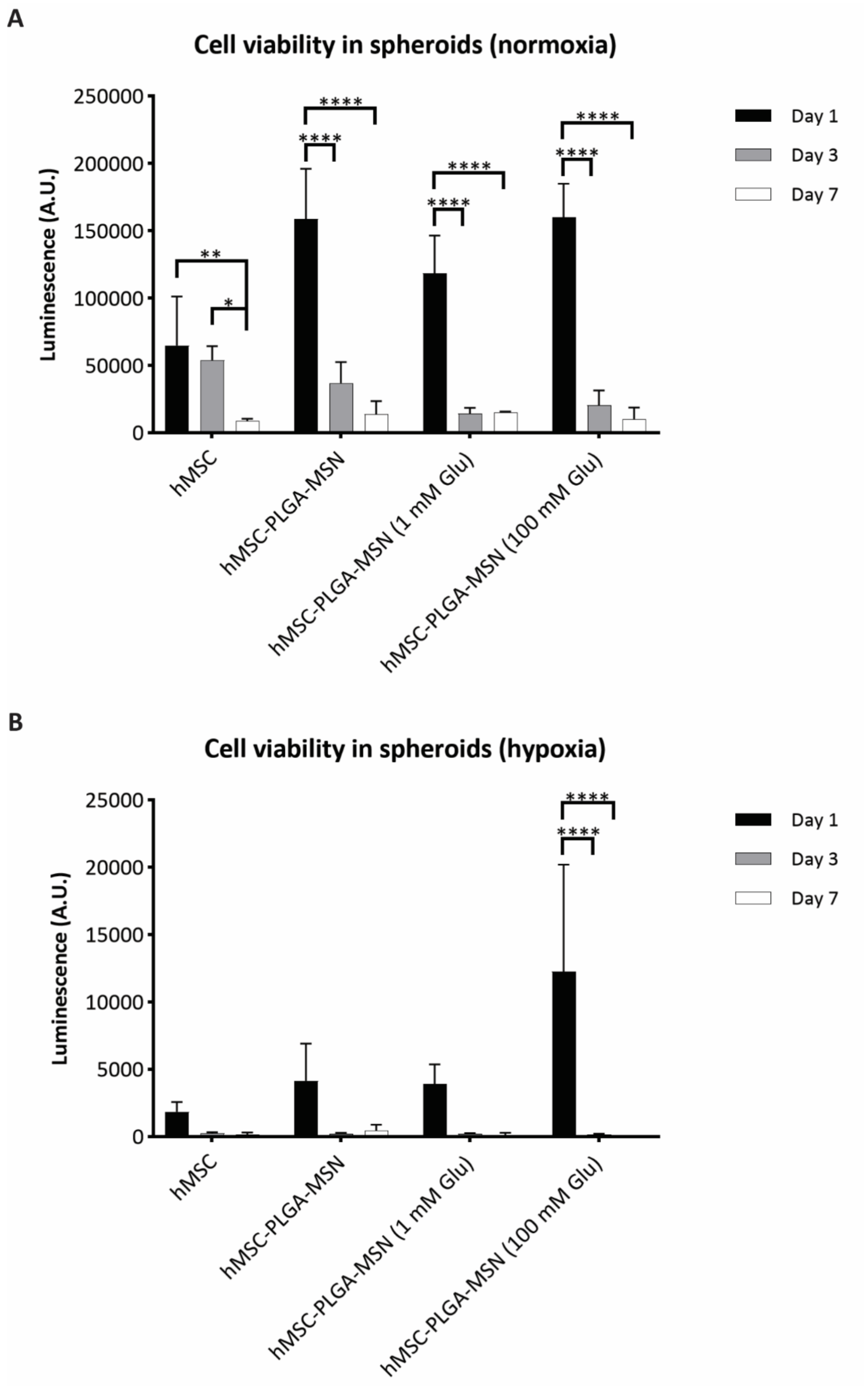


**Figure S8.** *Glucose effect on cell viability in hMSC*-nanofunctionalized microparticle *spheroids.* Quantification of cell viability in hMSC-nanofunctionalized microparticle spheroids in (A) normoxia and (B) hypoxia (5% $pO_2$) conditions after 1, 3 and 7 day of culture in DPBS by using the CellTiterGlo 3D Cell Viability Assay (n = 3). '*', '**', and '****' represents p values smaller than 0.05, 0.01, and 0.0001, respectively. A.U. stands for '*arbitrary units'.* Statistical method used is two-way ANOVA with Tukey's post hoc test for multiple comparisons.

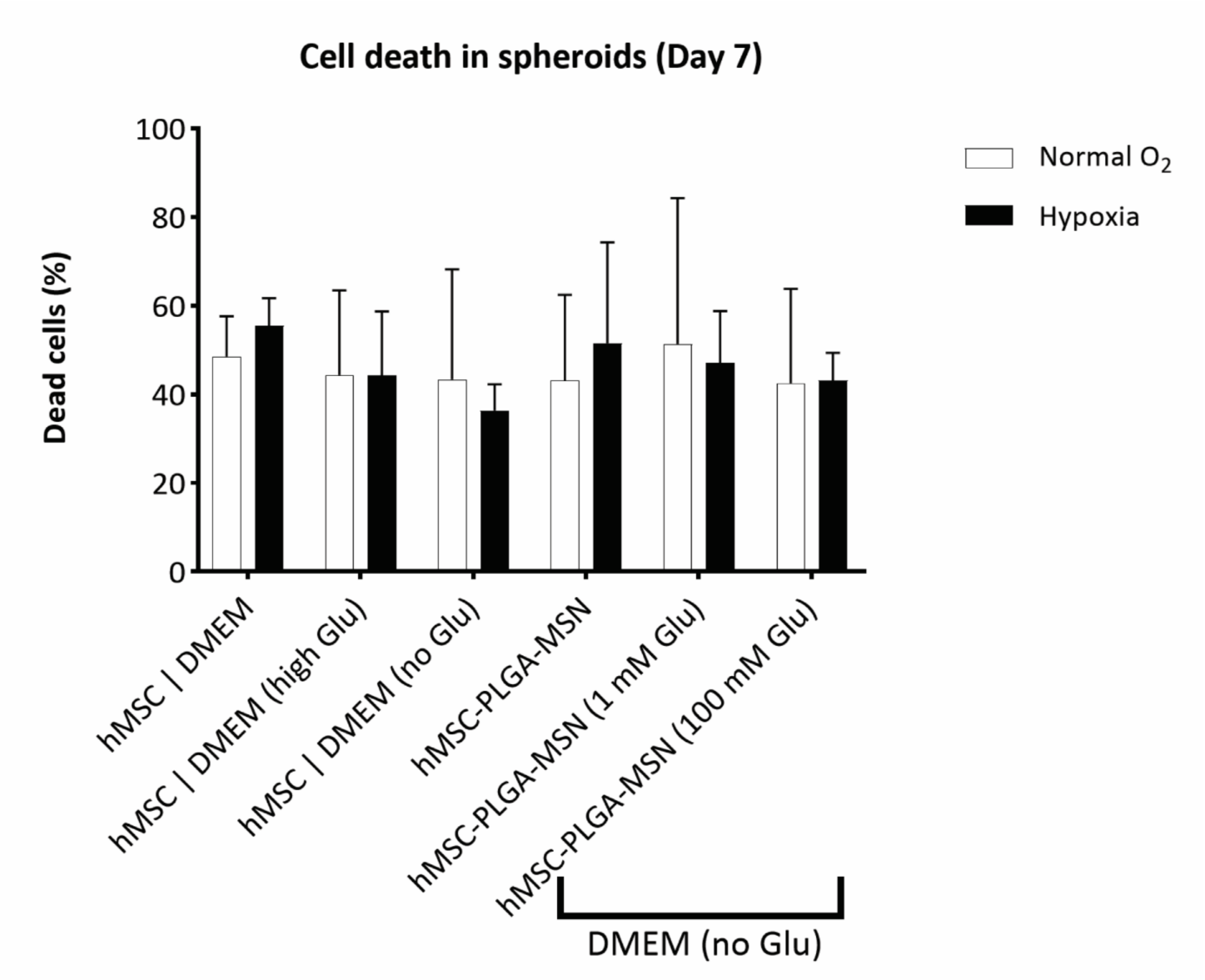


**Figure S9.** *Glucose release effect on cell death in hMSC*-nanofunctionalized microparticle *spheroids after 7 days in culture.* Quantification of cell viability in hMSC-nanofunctionalized microparticle spheroids in normoxia and hypoxia conditions (5% $pO_2$) after 7 days of culture by quantitative analysis of confocal fluorescence microscopy images in CellProfiler (n = 4). Statistical method used is two-way ANOVA with Tukey's post hoc test for multiple comparisons.

**Video S1.** *Aggregation of hMSC-PLGA-MSN spheroids over time.* Fluorescence microscopy live imaging of (A) hMSC spheroids, (B) hMSC-PLGA spheroids and (C) hMSC-PLGA-MSN spheroids over the course of 48 hours. The time lapses were created by every 30 min acquiring images of points of interest and exported as mp4 files for their visualization as videos. In each case, the cytoplasm was stained with CellTracker Green CMFDA (Thermo Fisher Scientific) (1.5 μM) (in green), while MSNs were labeled with ATTO647N-Maleimide (in red) (only in Video S1C).
